# GDF-15: a novel biomarker of heart failure predicts short-term and long-term heart-failure rehospitalization and short-term mortality in patients with acute heart failure syndrome

**DOI:** 10.1186/s12872-024-03802-5

**Published:** 2024-03-12

**Authors:** Paisit Kosum, Noppachai Siranart, Natthinee Mattanapojanat, Somkiat Phutinart, Narisorn Kongruttanachok, Supanee Sinphurmsukskul, Sarawut Siwamogsatham, Sarinya Puwanant, Aekarach Ariyachaipanich

**Affiliations:** 1 Division of Cardiovascular Medicine, Department of Medicine, Faculty of Medicine, Chulalongkorn University, Bangkok, Zip Code 10330 Thailand; 2 Division of Cardiovascular Medicine, Department of Medicine, Faculty of Medicine, Naresuan University, Phitsanulok, Thailand; 3 Department of Medicine, Faculty of Medicine, Chulalongkorn University, Bangkok, Thailand; 4 Division of Gastroenterology and Hepatology, Mayo Clinic, Rochester, MN USA; 5 Department of Laboratory Medicine, Faculty of Medicine, Chulalongkorn University, Bangkok, Thailand; 6 Excellent Center for Organ Transplantation, King Chulalongkorn Memorial Hospital, Thai Red Cross Society, Bangkok, Thailand; 7 Chula Clinical Research Center (ChulaCRC), Faculty of Medicine, Chulalongkorn University, Bangkok, Thailand; 8 Cardiac Center, King Chulalongkorn Memorial Hospital, Thai Red Cross Society, Bangkok, Thailand

**Keywords:** Acute heart failure syndrome, Biomarkers, GDF-15, Rehospitalization, All-cause mortality, Orthoedema congestion score

## Abstract

**Background:**

Acute heart failure (AHF) is a potentially life-threatening clinical syndrome, usually requiring hospital admission. Growth Differentiation Factor-15 (GDF-15) is a distant member of the transforming growth factor-β. The increased expression of GDF-15 has been observed during heart failure (HF) and is associated with worse outcomes. However, the relationship between GDF-15 and AHF is not well understood with limited evidence among Thai patients.

**Purpose:**

Investigate the correlation between biomarker levels (measured upon admission and discharge) and short- and long-term adverse outcomes, encompassing all-cause mortality and heart-failure (HF) rehospitalization (at 30, 90, and 180 days, as well as throughout the entire follow-up duration) in individuals experiencing acute HF.

**Methods:**

This is a prospective single-center investigation involving patients admitted for AHF. Biomarkers, including GDF-15, high-sensitivity troponin T (hsTnT), and N-terminal pro-B-type natriuretic peptide (NT-proBNP), were assessed upon admission and discharge. Outcomes, including all-cause mortality and HF rehospitalization, were examined. Logarithmic transformations were applied to the biomarker variables for subsequent analysis. Univariate and multivariate analyses of cause-specific hazards were conducted using the Cox proportional hazards regression model, while subdistribution hazards were assessed using the Fine-Gray regression model to evaluate outcomes.

**Results:**

A total of 84 patients were enrolled (mean age of 69 years, 52% females). The GDF-15 level significantly decreased during admission (median at the time of admission 6,346 pg/mL, median at the time of discharge 5,711 pg/mL; *p* < 0.01). All-cause mortality at 30 days and 180 days were 6.0% and 16.7%, respectively. HF rehospitalization at 30 days and 180 days were 15.5% and 28.6%, respectively. Univariate analysis showed that total orthoedema congestion score (*p* = 0.02) and admission GDF-15 level (*p* = 0.01) were associated with 30-day all-cause mortality, whereas hsTnT or NT-proBNP levels did not show significant associations. However, higher levels of NT-proBNP upon admission were associated with all-cause mortality when considering the entire follow-up period (*p* < 0.01). Both univariate and multivariate analyses demonstrated that lower discharge GDF-15 levels and a greater reduction in GDF-15 levels from admission to discharge were associated with a lower risk of 30-day rehospitalization. Similarly, univariate analysis revealed that a greater reduction in NT-proBNP levels from admission to discharge was associated with lower 30-day rehospitalization rates. At 180 days, a greater reduction in GDF-15 levels remained associated with lower hazards and incidence of rehospitalization.

**Conclusion:**

The significant decrease in Growth Differentiation Factor-15 (GDF-15) levels during hospitalization suggests its potential as a dynamic marker reflecting the course of AHF. Importantly, higher GDF-15 levels at admission were associated with an increased risk of 30-day all-cause mortality, highlighting its prognostic value in this patient population. Moreover, lower discharge GDF-15 levels, reductions in GDF-15 from admission to discharge, and decreases in NT-proBNP from admission to discharge were associated with a reduced risk of 30-day rehospitalization.

**Supplementary Information:**

The online version contains supplementary material available at 10.1186/s12872-024-03802-5.

## Introduction

Acute heart failure (AHF) represents a potentially life-threatening clinical syndrome often necessitating hospital admission. AHF may manifest de novo or in individuals with chronic heart failure (HF). There are global variations in the duration of AHF hospitalization. In numerous countries, patients are hospitalized for at least one week, experiencing an inpatient mortality rate ranging from 5–10%, and up to 25% of the patients are readmitted within a month of discharge [1].

Among patients readmitted within 30 days following discharge from an AHF hospitalization, 50% experienced relapses attributable to HF, while 23% stemmed from non-HF cardiovascular causes [2]. A quarter of patients hospitalized due to exacerbating HF exhibit signs of inadequate decongestion at discharge [3], and this is associated with an elevated risk of readmission and a worse prognosis [4]. In addition, congestion not only has a significant effect on symptoms and quality of life, but is also associated with cardiac, renal, and liver injury, which, in turn, are associated with adverse clinical outcome [5]. The gold standard for evaluating congestion in hospitalized patients the gold standard for assessing congestion in hospitalized patients involves measuring pulmonary capillary wedge pressure (PCWP) which provides a good approximation of left-ventricular filling pressure, allowing detection of hemodynamic congestion relatively early in the preclinical stage. However, PCWP measurement involves invasive catheterization, limiting its clinical utility, especially in outpatient settings. While there is currently no standardized definition of adequate decongestion, several criteria have been employed in clinical trials. These include jugular venous distention (JVD) < 8 cm of water, no more than trace peripheral edema, and the absence of orthopnea [6]. These criteria have been condensed into an orthoedema congestion score, with higher scores upon admission and discharge correlating with a heightened risk of adverse outcomes, including death or HF hospitalization within 60 days [7].

Over the past decade, there has been a surge of interest in the field of biomarkers concerning the management and care of patients with HF. New insights into the various facets of the intricate molecular interplay underlying HF have prompted the search for representative cardiac biomarkers with the anticipation that their combined use can help inform physicians of the patient’s disease state [8]. Among these biomarkers, natriuretic peptides, including B-type natriuretic peptide (BNP) and the N-terminal fragment of its prohormone (NT-proBNP), have emerged as approved biomarkers for HF [9]. Additionally, novel biomarkers such as ST-2, GDF-15, Pentraxin-3, Galectin-3, Osteopontin [10] have been the subject of investigation either individually or in combination within the HF context.

Growth-differentiation factor-15 (GDF-15) belongs to the transforming growth factor-β (TGF-β) cytokine superfamily and is primarily expressed the liver. It is also known as Prostate derived factor (PDF), Macrophage inhibitory cytokine-1(MIC-1), NSAID-activated gene (NAG-1) and Placental TGF-Beta (PTGFB). While the precise function of GDF-15 remains unclear, it is generally expressed at low levels across all tissue types under normal physiological states. However, increased expression of GDF-15 has been observed in various pathological states including pulmonary, cardiac, or renal diseases.

Multi-biomarkers (GDF-15, NT-proBNP, hsTnT) and orthoedema congestion score are rarely studied in Asian. Consequently, this study sought to examine the association between the multi-biomarker profile and congestion levels measured at admission and discharge, in relation to the outcomes of all-cause mortality and HF rehospitalization at 30, 90, and 180 days, as well as over the entire follow-up period, among patients diagnosed with AHF syndrome.

## Methodology and methods

### Study design and studied population

This is a prospective, single center study of patients admitted at King Chulalongkorn Memorial Hospital (KCMH) for AHF syndrome regardless of ejection fraction (acute de novo heart failure, acute decompensated heart failure) between December 2018 to June 2019. All patients were symptomatic (New York Heart Association [NHYA] functional class II-IV). The key inclusion criteria were as follows: 1. Adults aged over 18 years; 2. Inpatient status at the time of screening; 3. Admission diagnosis of AHF per primary; accepted terms include heart failure (HF), acute decompensated heart failure (ADHF), congestive heart failure; 4. Patient presence of at least 2 major criteria or 1 major criteria in conjunction with 2 minor criteria of Framingham criteria for congestive heart failure. Patients were excluded from the study if they were confused or in a delirium state, diagnosed with liver cirrhosis or nephrotic syndrome, had unacceptable vision or hearing impairments preventing them from correctly responding to the questionnaire, were unwilling to be reached by phone, were outpatients, or did not provide consent.

Our study protocol complied with the ethical guidelines of the 1975 Declaration of Helsinki and was conducted under the approval of the Institutional Review Board of the Research Ethics Review Committee for Research Involving Human Research Participants, Health Sciences Group, Chulalongkorn University (198/61). We obtained informed consent from all of the enrolled patients. Out study was registered in Thai Clinical Trials Registry (TCTR20190521002, Date of first registration: 21/05/2019).

### Data collection and data analysis

After screening inclusion and exclusion criteria, the enrolled patients underwent optimization of medical therapy as determined by their treating primary physician or HF cardiologist. We collected demographic data, previous left-ventricular ejection fraction (LVEF), co-morbidities such as diabetic mellitus (DM), coronary arterial disease (CAD), prior percutaneous coronary intervention (PCI) including stents and balloon angioplasty, coronary artery bypass grafting (CABG), atrial fibrillation (AF), and hypertension (HTN). General physical and cardiovascular examinations, including vital signs, jugular venous distention (JVD), rales or crepitations, and peripheral edema, were documented. Details regarding treatment, including length of stay, pharmacological interventions, diagnostic investigations, and interventions, were recorded using a case record form (CRF).

The orthoedema congestion score is determined based on the presence of orthopnea (≥ 2 pillows = 2, < 2 pillows = 0) and peripheral edema (trace: pitting edema grade 0 or 1 +  = 0, moderate: pitting edema grade 2 +  = 1, severe: pitting edema grade 3 + or 4 +  = 2), with the components combined to classify congestion as no congestion (score 0), low grade (score 1–2), or high grade (score 3–4).

After obtaining informed consent from the patients to participate in the study, the researcher collected a 3 ml blood specimen (in a lithium heparin tube) within 24 h after admission and 24 h prior to discharge (totaling 6 ml of blood specimen) for further biomarker investigations (GDF-15, NT-proBNP, and hsTnT). The specimens were analyzed for GDF-15, hsTnT, and NT-proBNP using the Cobas® Machine (Roche Diagnostics, Basel, Switzerland).

Following discharge, patients were contacted by phone 30 days after the discharge date to ascertain outcomes, including mortality, rehospitalization, the number and causes of rehospitalizations, and recent NYHA status.

### Outcomes

The primary objective of the study was to assess the association between biomarkers (GDF-15, NT-proBNP, and hsTnT) in patients with AHF and their correlation with 30-day HF rehospitalization or 30-day all-cause mortality. The secondary endpoints included examining the association between biomarkers in AHF patients and their correlation with HF rehospitalization or all-cause mortality at 90 and 180 days, as well as over the entire follow-up period.

### Statistical analysis

Logarithmic transformation was employed on all biomarker variables for subsequent analysis. To compare biomarker levels between discharge and baseline, log-transformed values (i.e., log_2_[admission] – log_2_[discharge]) were utilized, effectively representing the log-transformed ratio of biomarker levels at admission to discharge. Biomarker levels were considered both as continuous and categorical variables. For categorical biomarker variables, the biomarker values were stratified into two groups: low and high. The range for each biomarker variable's strata is provided in Table 1.

**Table 1:** Biomarker strata by median value

Biomarker strata	
**Factors**	**Range**
**NT-proBNP (pg/mL)**	
Low	≤ 4577.00
High	> 4577.00
**hsTnT (pg/mL)**	
Low	≤ 38.53
High	> 38.53
**GDF-15 (pg/mL)**	
Low	≤ 6221.50
High	> 6221.50
**NT-proBNP admission:discharge**	
Low	≤ 1.0992464
High	> 1.0992464
**hsTnT admission:discharge**	
Low	≤ 1.13408464
High	> 1.13408464
**GDF-15 admission:discharge**	
Low	≤ 1.8079528
High	> 1.8079528

All categorical and continuous variables underwent analysis using Fisher’s exact test and Welch’s T-test, respectively. When comparing paired biomarker levels between baseline and discharge, the Wilcoxon signed-rank test with continuity correction and McNemar’s Chi-squared test were employed for continuous and categorical variables, respectively. For other comparisons, the Wilcoxon rank-sum test and Fisher’s exact test were utilized for continuous and categorical variables, respectively. Continuous variables were presented as both mean and standard deviation, as well as median and interquartile range, while categorical variables were expressed as absolute count and relative proportion.

Univariate and multivariate analyses of cause-specific hazards were conducted using the Cox proportional hazards regression model to examine the relationship between biomarkers and HF rehospitalization, with all-cause mortality considered as a competing risk. Subdistribution hazards were analyzed using the Fine-Gray regression model. Patients with incomplete data are excluded from a particular analysis only if that analysis requires the missing data. Otherwise, all other available data is included in the analysis.

For outcomes related to all-cause mortality, which did not involve competing risks, univariate analysis was performed using the Cox proportional hazards regression model for hazard ratio estimation, and the log-rank test was utilized to test for differences between survival curves. Multivariable analysis for all-cause mortality was conducted using cause-specific hazards analysis with the Cox proportional hazards regression model.

These analyses were conducted for both categorical and continuous biomarker covariates. These analyses encompassed both categorical and continuous biomarker covariates. Hazard ratios were calculated using partial likelihood estimation within the fitted model. Confidence intervals for these hazard ratios were determined utilizing the Wald test. However, due to the relatively small sample size, likelihood ratio tests were employed to establish *p*-values for both continuous and binary categorical variables, as well as to compute the overall *p*-value for non-binary categorical variables when assessing these covariates in the survival models. *P*-values for individual levels within non-binary categorical variables were derived using the Wald test. For categorical variables with more than two levels, both the *p*-values comparing each level to the baseline and the overall *p*-values were reported.

In the multivariable analysis, only biomarker variables were included for analysis, as including too many covariates often resulted in non-convergence. For each outcome of interest and variable type (categorical or continuous biomarker variables), three sets of analyses were conducted on the three biomarker covariates (NT-proBNP, hs-TnT, and GDF-15) based on the timing and nature in which the covariates were obtained (i.e., level at admission, level at discharge, and the change from admission to discharge). This approach was adopted to mitigate potential collinearity issues when performing multivariable regression.

To assess whether the proportional hazards assumption holds for each covariate within each outcome in the Cox-proportional hazards regression model, Schoenfeld residuals were assessed for any significant relationship with time. A significant relationship for a covariate at a given time point indicates non-proportional hazards and may be contribute to varying degrees of association between covariates and outcomes at different time points.

All statistical analyses were conducted using the R Statistical Software (v4.3.2; R Core Team 2023). A *p*-value of < 0.05 was considered statistically significant.

## Results

**Fig. 1:**
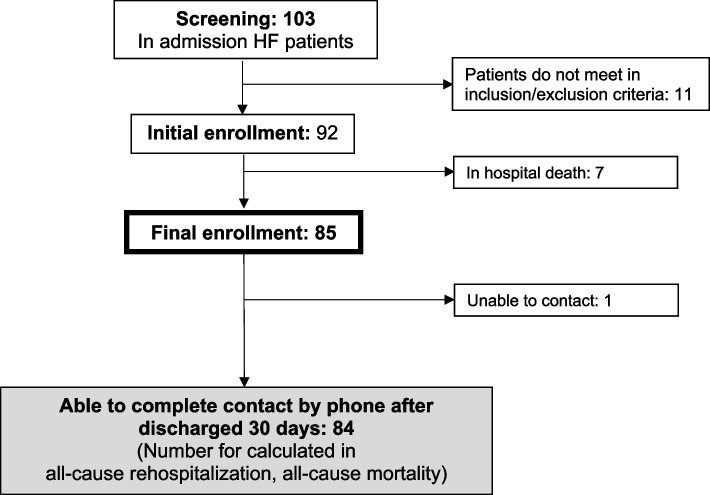
Patient CONSORT diagram

A total of 103 patients were initially screened, out of which 11 were excluded from the study due to incomplete data (6 did not meet the criteria for AHF, 2 were referred to a nearby hospital, and 3 refused to participate), leaving 92 patients who were enrolled in the study. During the course of the study, seven patients experienced intra-hospital mortality, while one patient was lost to follow-up due to inability to establish telephone contact. Consequently, a total of 84 patients were included in the final analysis (Fig. 1). The median follow-up time was 213 days.

### Baseline characteristics

The baseline characteristics of the study population, stratified into the 30-day readmission group and non-readmission group, are summarized in Table 2. The mean age of the cohort was 69 ± 14 years, with females comprising 52.4% of the participants. Ischemic heart disease was reported in 38.1% of cases, while 75% had hypertension, 59.5% had diabetes mellitus, 56% had dyslipidemia, 23.1% had a history of HF with reduced ejection fraction (EF) and 39.3% had chronic kidney disease, at least stage III. The mean jugular venous distention (JVD) was 9.0 ± 1.4 cm, and the mean body weight was 69.9 ± 22.0 kg.

**Table 2:** Baseline characteristics of patients diagnosed with acute heart failure syndrome with 30-day HF rehospitalization

Factors	Total (*N* = 84)	Non rehospitalization (*N* = 71)	Rehospitalization (*N* = 13)	*P* value
**Age** (mean ± S.D.)	69 ± 14 (19–97)	70 ± 13 (34–97)	66 ± 20 (19–92)	0.535
**Male gender**	40 (47.6%)	36 (50.7%)	4 (30.8%)	0.186
**Ethnicity** (Thai)	83 (98.8%)	70 (98.6%)	13 (100%)	1.000
**Past History**				
Hx of HF	60 (73.2%)	48 (69.6%)	12 (92.3%)	0.169
LVEF				0.310
No previous echo	11 (16.9%)	11 (20.4%)	0	
pEF (> = 50%)	34 (52.3%)	28 (51.9%)	6 (54.5%)	
mrEF (40–49%)	5 (7.7%)	4 (7.4%)	1 (9.1%)	
rEF (< 40%)	15 (23.1%)	11 (20.4%)	4 (36.4%)	
Hx of HF hospitalization	44 (67.7%)	36 (64.3%)	8 (88.9%)	0.251
Diabetes mellitus	50 (59.5%)	44 (62%)	6 (46.2%)	0.285
Ischemic heart disease	32 (38.1%)	26 (36.6%)	6 (46.2%)	0.546
Atrial fibrillation	27 (32.1%)	23 (32.4%)	4 (30.8%)	1.000
Hypertension	63 (75%)	57 (80.3%)	6 (46.2%)	0.015
Dyslipidemia	47 (56%)	43 (60.6%)	4 (30.8%)	0.047
CKD at least stage III	33 (39.3%)	30 (42.2%)	3 (23.1%)	0.362
**Treatment (Prior to admission) (mean ± S.D.)/median (IQR)**				
Furosemide	51 (60.7%)	40 (56.3%)	11 (84.6%)	0.055
Furosemide (mg/day)	171.57 ± 272.78 40 (20–240)	145 ± 237.17 40 (20–120)	268.18 ± 373.60 60 (40–250)	0.327
Betablocker (BB)	49 (58.3%)	41 (57.7%)	8 (61.5%)	0.799
ACEI	11 (13.1%)	10 (14.1%)	1 (7.7%)	1.000
ARB	15 (17.9%)	13 (18.3%)	2 (15.4%)	1.000
Spironolactone	7 (8.3%)	5 (7%)	2 (15.4%)	0.295
Digoxin	5 (6%)	3 (4.2%)	2 (15.4%)	0.169
**Treatment at discharge (mean ± S.D.)/median (IQR)**				
Furosemide	66 (78.6%)	54 (76.1%)	12 (92.3%)	0.281
Furosemide (mg/day)	190.92 ± 276.18 80 (40–160)	174.34 ± 269.93 80 (20–160)	264.17 ± 303.6 120 (40–500)	0.210
HCTZ	2 (2.4%)	1 (1.4%)	1 (7.7%)	0.287
Tolvaptan	1 (1.2%)	0	1 (7.7%)	0.155
Betablocker	50 (59.5%)	42 (59.2%)	8 (61.5%)	0.872
ACEI	9 (10.7%)	8 (11.3%)	1 (7.7%)	1.000
ARB	8 (9.5%)	8 (11.3%)	0	0.347
Spironolactone	4 (4.8%)	2 (2.8%)	2 (15.4%)	0.111
Digoxin	5 (6%)	4 (5.6%)	1 (7.7%)	0.578

Patients in the non-readmission group exhibited a higher prevalence of hypertension and dyslipidemia compared to the rehospitalization group (80.3% vs. 46.2%; *p* = 0.015 and 60.6% vs. 30.8%; *p* = 0.047, respectively). However, no significant differences were observed in terms of age, sex, left ventricular ejection fraction (LVEF), number of prior cardiovascular diseases, history of HF hospitalization, or prehospital drug usage (including diuretics, beta-blockers, ACEI/ARB/ARNI, digoxin, ivabradine, hydralazine, nitrates, anticoagulants, and P2Y12 inhibitors).

Interestingly, patients in the rehospitalization group had a lower rate of statin usage both prior to admission (23.1% vs. 73.2%; *p* = 0.001) and at discharge (30.8% vs. 66.2%; *p* = 0.016).

### Orthoedema congestion score

The orthoedema congestion score of the study population is detailed in Tables 3, 4, 5 and 6 and Supplementary Table S1-S12.

**Table 3:** Orthoedemascore and continuous biomarker covariates in patients with 30-day HF rehospitalization

**30-day heart failure rehospitalization**				
**Admission**				
**Factors**	**Total**	**Non rehospitalization**	**Rehospitalization**	***P* value**
	**(*N* = 84)**	**(*N* = 71)**	**(*N* = 13)**	
**Total orthoedema score**				0.64
mean ± S.D	2.29 ± 0.98	2.31 ± 1.01	2.15 ± 0.80	
median (IQR)	2.00 (2.00–3.00)	2.00 (2.00–3.00)	2.00 (2.00–3.00)	
**NT-proBNP (pg/mL)**				0.30
mean ± S.D	9,901 ± 10,132	10,505 ± 10,477	6,601 ± 7,470	
median (IQR)	5,529 (2,260–13,918)	5,628 (2,283–15,889)	3,513 (2,272–8,896)	
**hsTnT (pg/mL)**				0.11
mean ± S.D	217 ± 852	245 ± 923	67 ± 125	
median (IQR)	40 (27–94)	44 (27–100)	32 (22–34)	
**Factors**	**Total**	**Non rehospitalization**	**Rehospitalization**	***P* value**
	**(*N* = 83)**	**(*N* = 71)**	**(*N* = 12)**	
**GDF-15 (pg/mL)**				0.91
mean ± S.D	9,119 ± 6,011	9,318 ± 6,366	7,942 ± 3,111	
median (IQR)	6,879 (4,428–12,387)	6,855 (4,132–13,290)	7,330 (5,344–11,013)	
**Discharge**				
**Factors**	**Total**	**Non rehospitalization**	**Rehospitalization**	***P* value**
	**(*N* = 84)**	**(*N* = 71)**	**(*N* = 13)**	
**Total orthoedema score**				0.053
mean ± S.D	0.38 ± 0.79	0.30 ± 0.72	0.77 ± 1.01	
median (IQR)	0.00 (0.00–0.00)	0.00 (0.00–0.00)	0.00 (0.00–2.00)	
**Factors**	**Total**	**Non rehospitalization**	**Rehospitalization**	***P* value**
	**(*N* = 71)**	**(*N* = 60)**	**(*N* = 11)**	
**Log NT-proBNP (pg/mL)**				0.28
mean ± S.D	7,091 ± 11,041	7,298 ± 11,870	5,961 ± 4,514	
median (IQR)	3,306 (690–7,863)	2,862 (671–7,447)	4,739 (3,140–9,235)	
**hsTnT (pg/mL)**				0.64
mean ± S.D	92 ± 165	97 ± 173	67 ± 110	
median (IQR)	37 (22–64)	38 (20–69)	28 (26–46)	
**GDF-15 (pg/mL)**				0.028
mean ± S.D	7,107 ± 4,788	6,737 ± 4,893	9,128 ± 3,728	
median (IQR)	5,659 (3,662–8,735)	5,376 (3,370–8,102)	8,539 (6,898–10,911)	
**NT-proBNP admission:discharge**				0.002
mean ± S.D	2.88 ± 3.84	3.20 ± 4.09	1.16 ± 0.92	
median (IQR)	1.81 (1.08–3.04)	1.96 (1.37–3.06)	0.69 (0.61–1.39)	
**hsTnT admission:discharge**				0.19
mean ± S.D	1.48 ± 1.43	1.46 ± 1.35	1.56 ± 1.89	
median (IQR)	1.13 (0.96–1.34)	1.14 (1.00–1.35)	0.95 (0.85–1.18)	
**Factors**	**Total**	**Non rehospitalization**	**Rehospitalization**	***P* value**
	**(*N* = 70)**	**(*N* = 60)**	**(*N* = 10)**	
**GDF-15 admission:discharge**				0.018
mean ± S.D	1.39 ± 0.93	1.47 ± 0.97	0.89 ± 0.38	
median (IQR)	1.10 (0.96–1.63)	1.20 (1.00–1.66)	0.95 (0.70–1.09)	

*P* value by Wilcoxon rank sum test, Welch Two Sample t-test

**Table 4:** Orthoedemascore and categorical biomarker covariates in patients with 30-day HF rehospitalization

**30-day heart failure rehospitalization**				
**Admission**				
**Factors**	**Total**	**Non rehospitalization**	**Rehospitalization**	***P* value**
	**(*N* = 84)**	**(*N* = 71)**	**(*N* = 13)**	
**Grading orthoedema score**				0.90
No congestion (0)	6 (7.1%)	5 (7.0%)	1 (7.7%)	
Low grade (1–2)	47 (56%)	39 (55%)	8 (62%)	
High grade (3–4)	31 (37%)	27 (38%)	4 (31%)	
**NT-proBNP (pg/mL)**				0.55
Low (≤ 4577.00)	37 (44%)	30 (42%)	7 (54%)	
High (> 4577.00)	47 (56%)	41 (58%)	6 (46%)	
**hsTnT (pg/mL)**				0.033
Low (≤ 38.53)	40 (48%)	30 (42%)	10 (77%)	
High (> 38.53)	44 (52%)	41 (58%)	3 (23%)	
**Factors**	**Total**	**Non rehospitalization**	**Rehospitalization**	***P* value**
	**(*N* = 83)**	**(*N* = 71)**	**(*N* = 12)**	
**GDF-15 (pg/mL)**				> 0.99
Low (≤ 6221.50)	38 (46%)	33 (46%)	5 (42%)	
High (> 6221.50)	45 (54%)	38 (54%)	7 (58%)	
**Discharge**				
**Factors**	**Total**	**Non rehospitalization**	**Rehospitalization**	***P*** **value**
	**(*N* = 84)**	**(*N* = 71)**	**(*N* = 13)**	
**Grading orthoedema score**				0.11
No congestion (0)	64 (81%)	56 (85%)	8 (62%)	
Congestion present (≥ 1)	15 (19%)	10 (15%)	5 (38%)	
**Factors**	**Total**	**Non rehospitalization**	**Rehospitalization**	***P*** **value**
	**(*N* = 71)**	**(*N* = 60)**	**(*N* = 11)**	
**NT-proBNP (pg/mL)**				0.51
Low (≤ 4577.00)	41 (58%)	36 (60%)	5 (45%)	
High (> 4577.00)	30 (42%)	24 (40%)	6 (55%)	
**hsTnT (pg/mL)**				0.20
Low (≤ 38.53)	38 (54%)	30 (50%)	8 (73%)	
High (> 38.53)	33 (46%)	30 (50%)	3 (27%)	
**GDF-15 (pg/mL)**				0.018
Low (≤ 6221.50)	39 (55%)	37 (62%)	2 (18%)	
High (> 6221.50)	32 (45%)	23 (38%)	9 (82%)	
**NT-proBNP admission:discharge**				0.046
Low (≤ 1.0992464)	36 (51%)	27 (45%)	9 (82%)	
High (> 1.0992464)	35 (49%)	33 (55%)	2 (18%)	
**hsTnT admission:discharge**				> 0.99
Low (≤ 1.13408464)	36 (51%)	30 (50%)	6 (55%)	
High (> 1.13408464)	35 (49%)	30 (50%)	5 (45%)	
**Factors**	**Total**	**Non rehospitalization**	**Rehospitalization**	***P* value**
	**(*N* = 70)**	**(*N* = 60)**	**(*N* = 10)**	
**GDF-15 admission:discharge**				0.31
Low (≤ 1.8079528)	35 (50%)	28 (47%)	7 (70%)	
High (> 1.8079528)	35 (50%)	32 (53%)	3 (30%)	

*P* value by Fisher's exact test

**Table 5:** Orthoedemascore and continuous biomarker covariates in patients with 30-day all-cause mortality

**30-day all-cause mortality**				
**Admission**				
**Factors**	**Total**	**Alive**	**Deaths**	***P* value**
	**(*N* = 84)**	**(*N* = 79)**	**(*N* = 5)**	
**Total orthoedema score**				0.026
mean ± S.D	2.29 ± 0.98	2.23 ± 0.96	3.20 ± 0.84	
median (IQR)	2.00 (2.00–3.00)	2.00 (2.00–3.00)	3.00 (3.00–4.00)	
**NT-proBNP (pg/mL)**				0.22
mean ± S.D	9,901 ± 10,132	9,394 ± 9,680	17,915 ± 14,753	
median (IQR)	5,529 (2,260–13,918)	5,520 (2,247–12,031)	19,629 (4,808–28,992)	
**hsTnT (pg/mL)**				0.045
mean ± S.D	217 ± 852	217 ± 876	223 ± 262	
median (IQR)	40 (27–94)	39 (26–84)	151 (52–197)	
**Factors**	**Total**	**Alive**	**Deaths**	***P* value**
	**(*N* = 83)**	**(*N* = 78)**	**(*N* = 5)**	
**GDF-15 (pg/mL)**				0.012
mean ± S.D	9,119 ± 6,011	8,702 ± 5,886	15,619 ± 4,175	
median (IQR)	6,879 (4,428–12,387)	6,346 (4,206–11,734)	14,297 (12,880–20,000)	
**Discharge**				
**Factors**	**Total**	**Non rehospitalization**	**Rehospitalization**	***P* value**
	**(*N* = 79)**	**(*N* = 78)**	**(*N* = 1)**	
**Total orthoedema score**				0.65
mean ± S.D	0.38 ± 0.79	0.38 ± 0.79	0.00 ± NA	
median (IQR)	0.00 (0.00–0.00)	0.00 (0.00–0.00)	0.00 (0.00–0.00)	
**Factors**	**Total**	**Non rehospitalization**	**Rehospitalization**	***P* value**
	**(*N* = 71)**	**(*N* = 70)**	**(*N* = 1)**	
**Log NT-proBNP (pg/mL)**				0.23
mean ± S.D	7,091 ± 11,041	7,185 ± 11,092	479 ± NA	
median (IQR)	3,306 (690–7,863)	3,447 (701–8,011)	479 (479–479)	
**hsTnT (pg/mL)**				0.45
mean ± S.D	92 ± 165	93 ± 166	60 ± NA	
median (IQR)	37 (22–64)	36 (22–65)	60 (60–60)	
**GDF-15 (pg/mL)**				0.16
mean ± S.D	7,107 ± 4,788	6,999 ± 4,734	14,668 ± NA	
median (IQR)	5,659 (3,662–8,735)	5,632 (3,658–8,531)	14,668 (14,668–14,668)	
**NT-proBNP admission:discharge**				0.57
mean ± S.D	2.88 ± 3.84	2.89 ± 3.87	2.39 ± NA	
median (IQR)	1.81 (1.08–3.04)	1.79 (1.06–3.05)	2.39 (2.39–2.39)	
**hsTnT admission:discharge**				0.23
mean ± S.D	1.48 ± 1.43	1.48 ± 1.44	0.87 ± NA	
median (IQR)	1.13 (0.96–1.34)	1.14 (0.97–1.34)	0.87 (0.87–0.87)	
**Factors**	**Total**	**Non rehospitalization**	**Rehospitalization**	***P* value**
	**(*N* = 70)**	**(*N* = 69)**	**(*N* = 1)**	
**GDF-15 admission:discharge**				0.15
mean ± S.D	1.39 ± 0.93	1.40 ± 0.94	0.74 ± NA	
median (IQR)	1.10 (0.96–1.63)	1.10 (0.96–1.64)	0.74 (0.74–0.74)	

*P* value by Wilcoxon rank sum test, Welch Two Sample t-test

**Table 6:** Orthoedemascore and categorical biomarker covariates in patients with 30-day all-cause mortality

**30-day all-cause mortality**				
**Admission**				
**Factors**	**Total**	**Alive**	**Deaths**	***P* value**
	**(*N* = 84)**	**(*N* = 79)**	**(*N* = 5)**	
**Grading orthoedema score**				0.17
No congestion (0)	6 (7.1%)	6 (7.6%)	0 (0%)	
Low grade (1–2)	47 (56%)	46 (58%)	1 (20%)	
High grade (3–4)	31 (37%)	27 (34%)	4 (80%)	
**NT-proBNP (pg/mL)**				0.38
Low (≤ 4577.00)	37 (44%)	36 (46%)	1 (20%)	
High (> 4577.00)	47 (56%)	43 (54%)	4 (80%)	
**hsTnT (pg/mL)**				0.36
Low (≤ 38.53)	40 (48%)	39 (49%)	1 (20%)	
High (> 38.53)	44 (52%)	40 (51%)	4 (80%)	
**Factors**	**Total**	**Alive**	**Deaths**	***P* value**
	**(*N* = 83)**	**(*N* = 78)**	**(*N* = 5)**	
**GDF-15 (pg/mL)**				0.059
Low (≤ 6221.50)	38 (46%)	38 (49%)	0 (0%)	
High (> 6221.50)	45 (54%)	40 (51%)	5 (100%)	
**Discharge**				
**Factors**	**Total**	**Non rehospitalization**	**Rehospitalization**	***P* value**
	**(*N* = 79)**	**(*N* = 78)**	**(*N* = 1)**	
**Grading orthoedema score**				> 0.99
No congestion (0)	64 (81%)	63 (81%)	1 (100%)	
Congestion present (≥ 1)	15 (19%)	15 (19%)	0 (0%)	
**Factors**	**Total**	**Non rehospitalization**	**Rehospitalization**	***P* value**
	**(*N* = 71)**	**(*N* = 70)**	**(*N* = 1)**	
**NT-proBNP (pg/mL)**				> 0.99
Low (≤ 4577.00)	41 (58%)	40 (57%)	1 (100%)	
High (> 4577.00)	30 (42%)	30 (43%)	0 (0%)	
**hsTnT (pg/mL)**				0.46
Low (≤ 38.53)	38 (54%)	38 (54%)	0 (0%)	
High (> 38.53)	33 (46%)	32 (46%)	1 (100%)	
**GDF-15 (pg/mL)**				0.45
Low (≤ 6221.50)	39 (55%)	39 (56%)	0 (0%)	
High (> 6221.50)	32 (45%)	31 (44%)	1 (100%)	
**NT-proBNP admission:discharge**				0.49
Low (≤ 1.0992464)	36 (51%)	36 (51%)	0 (0%)	
High (> 1.0992464)	35 (49%)	34 (49%)	1 (100%)	
**hsTnT admission:discharge**				> 0.99
Low (≤ 1.13408464)	36 (51%)	35 (50%)	1 (100%)	
High (> 1.13408464)	35 (49%)	35 (50%)	0 (0%)	
**Factors**	**Total**	**Non rehospitalization**	**Rehospitalization**	***P* value**
	**(*N* = 70)**	**(*N* = 69)**	**(*N* = 1)**	
**GDF-15 admission:discharge**				> 0.99
Low (≤ 1.8079528)	35 (50%)	34 (49%)	1 (100%)	
High (> 1.8079528)	35 (50%)	35 (51%)	0 (0%)	

*P* value by Fisher's exact test

At the time of admission, the orthoedema congestion score was 2.29 ± 0.98. The grading of orthoedema congestion score consisted of no congestion (score 0) in 7.1% of patients, low grade (score 1–2) in 56% of patients, and high grade (score 3–4) in 36.9% of patients.

Upon discharge, the orthoedema congestion score was 0.38 ± 0.79. The grading of orthoedema congestion score comprised no congestion (score 0) in 81% of patients, low grade (score 1–2) in 19% of patients, and no patients with high grade (score 3–4) congestion.

Notably, there were no significant differences observed in the orthoedema congestion score between patients with AHF who experienced 30-day HF rehospitalization and those who encountered 30-day all-cause mortality.

### Rehospitalization and all-cause mortality

The highest rate of readmission occurred within the first 30 days after discharge, with a median time to readmission of 12 days. Death from any cause at 30 days was recorded in 5 patients (6.0%), while 30-day HF rehospitalization occurred in 13 patients (15.5%). By 90 days, 19 patients (22.6%) had been readmitted for HF, and all-cause mortality occurred in 8 patients (9.5%). At 180 days, HF rehospitalization occurred in 24 patients (28.6%), and a total of 14 patients had died (16.7%). At the final follow-up period, 25 patients (29.8%) had been readmitted due to HF, and 21 patients (25.0%) had died from any cause.

### Biomarkers

The biomarkers of the study population are presented in Tables 3, 4, 5 and 6, Supplementary Table S1-S12.

All biomarkers were significantly higher at admission compared to baseline. The median NT-proBNP level was 5182 pg/mL (IQR 2104–11667) at admission, compared to 7142 pg/mL (IQR 684–8011) at discharge (*p* < 0.01). Similarly, for hsTnT, the median level at admission was 42 pg/mL (IQR 25–84), which decreased to 37 pg/mL (IQR 22–65) at discharge (*p* < 0.01). Finally, the median GDF-15 level was 6346 pg/mL (IQR 4408–11460) at baseline and declined to 5711 pg/mL (IQR 3658–8832) at discharge (*p* < 0.01).

#### 30-day all-cause mortality

For 30-day all-cause mortality (Tables 7 and 8), univariate analysis showed that both a higher total orthoedema score (HR 3.18, CI 1.09–9.33) and elevated GDF-15 levels (HR 4.57, CI 1.11–18.91) upon admission were associated with increased hazard and incidence of death. This finding was further supported test which yielded significant result for GDF-15 level at admission (*p* = 0.03), suggesting that patients with different GDF-15 levels at baseline have distinct survival distributions.

**Table 7:** Survival analysis for 30-day all-cause mortality using continuous covariates

30-day all-cause mortality						
**Factor**	**Univariate analysis**			**Multivariate analysis**		
	**Cox proportional hazards**		**Schoenfeld residuals**	**Cox proportional hazards**		**Schoenfeld residuals**
	**HR (95%CI)**	***p*-value**	***p*-value**	**HR (95%CI)**	***p*-value**	***p*-value**
**Admission**						
**Total orthoedema score**	3.18 (1.09–9.33)	**0.02**	0.27			
**Log NT-proBNP (pg/mL)**	1.44 (0.8–2.58)	0.20	**0.02**	0.92 (0.43–1.99)	0.83	**0.03**
**Log hsTnT (pg/mL)**	1.32 (0.93–1.85)	0.16	0.57	1.22 (0.74–1.99)	0.45	0.54
**Log GDF-15 (pg/mL)**	4.57 (1.11–18.91)	** < 0.01**	0.41	4.42 (0.96–20.3)	**0.02**	0.41
**Discharge**						
**Total orthoedema score**	NA	0.52				
**Log NT-proBNP (pg/mL)**	0.6 (0.21–1.67)	0.27		NA	**0.02**	0.43
**Log hsTnT (pg/mL)**	1.18 (0.37–3.75)	0.78		NA	1.00	0.94
**Log GDF-15 (pg/mL)**	8.4 (0.23–300.48)	0.12		NA	**0.03**	0.99
**Diff Log NT-proBNP (pg/mL)**	1.23 (0.28–5.36)	0.78		1.51 (0.42–5.44)	0.55	0.25
**Diff Log hsTnT (pg/mL)**	0.59 (0.11–3.17)	0.60		0.92 (0.16–5.22)	0.92	0.62
**Diff Log GDF-15 (pg/mL)**	0.23 (0.02–3.09)	0.30		0.17 (0.00–6.43)	0.33	0.49

**Table 8:** Survival analysis for 30-day all-cause mortality using categorical covariates

30-day all-cause mortality								
**Factor**	**Threshold**	**Univariate analysis**				**Multivariate analysis**		
		**Cox proportional hazards**		**Log-Rank**	**Schoenfeld residuals**	**Cox proportional hazards**		**Schoenfeld residuals**
		**HR (95%CI)**	***p*-value**	***p*-value**	***p*-value**	**HR (95%CI)**	***p*-value**	***p*-value**
**Admission**								
**Grading orthoedema score**			0.12	0.12	0.85			
No congestion	0	1.00 (reference)						
Low grade	1–2	13310950 (0-Inf)	1.00	1.00				
High grade	3–4	83623350 (0-Inf)	1.00	1.00				
**NT-proBNP**	> 4577.00 pg/mL	3.31 (0.37–29.6)	0.24	0.26	**0.03**	1.02 (0.06–16.3)	0.99	**0.03**
**hsTnT**	> 38.53 pg/mL	3.7 (0.41–33.1)	0.19	0.21	0.58	2.68 (0.17–43.1)	0.49	0.58
**GDF-15**	> 6221.50 pg/mL	531526700 (0-Inf)	**0.01**	**0.03**	1.00	460,058,863 (0.00-Inf)	1.00	1.00
**Discharge**								
**Grading orthoedema score**	≥ 1 (congestion present)	0 (0-Inf)	0.52	0.63				
**NT-proBNP**	> 4577.00 pg/mL	0 (0-Inf)	0.29	0.39		0.00 (0.00-Inf)	1.00	0.79
**hsTnT**	> 38.53 pg/mL	690142100 (0-Inf)	0.22	0.28		16,794,936,545 (0.00-Inf)	1.00	0.73
**GDF-15**	> 6221.50 pg/mL	736669700 (0-Inf)	0.21	0.27		3,593,508,140 (0.00-Inf)	1.00	0.77
**NT-proBNP admission:discharge**	> 1.0992464	611477,700 (0-Inf)	0.23	0.31		1,679,461,779 (0.00-Inf)	1.00	0.82
**hsTnT admission:discharge**	> 1.13408464	0 (0-Inf)	0.24	0.32		0.00 (0.00-Inf)	1.00	0.92
**GDF-15 admission:discharge**	> 1.8079528	0 (0-Inf)	0.24	0.32		0.00 (0.00-Inf)	1.00	0.57

Moreover, GDF-15 and NT-proBNP levels at discharge also exhibited a significant association with hazards and incidence of death in the multivariate analysis (*p* = 0.03 and *p* = 0.02, respectively). However, the current analysis was not able to quantify this relationship due to non-convergence, likely attributable to the limited number of deaths within the 30-day period. Additionally, the absence of a significant association for these covariates in the univariate analysis may suggest potential interaction between them.

#### 30-day HF rehospitalization

For 30-day HF rehospitalization (Tables 9 and 10), GDF-15 level at discharge was significantly associated with increased hazard and incidence of 30-day HF readmission in both univariate (_CS_HR 1.92, CI 1.01–3.65; _SD_HR 1.92, CI 1.18–3.13) and multivariate analyses (_CS_HR 2.45, CI 1.75–5.13; _SD_HR 2.43, CI 1.26–4.71). Using a threshold of 6221.50 pg/mL, the group with higher level upon discharge also exhibited significant association with increased hazards and incidence of readmission at 30 days in both univariate (_CS_HR 6.30, CI 1.36–29.20; _SD_HR 6.30, CI 1.40–28.31) and multivariate analyses (_CS_HR 8.22, CI 1.70–39.70; _SD_HR 8.24, CI 1.68–40.40). Interestingly, decrease in GDF-15 levels from admission to discharge was also associated with decreased hazards and incidence of 30-day HF rehospitalization in both univariate (_CS_HR 0.17, CI 0.06–0.46; _SD_HR 0.17, CI 0.08–0.36) and multivariate analyses (_CS_HR 0.25, CI 0.08–0.78; _SD_HR 0.25, CI 0.10–0.65).

**Table 9:** Survival analysis for 30-day HF rehospitalization using continuous covariates

30-day heart failure rehospitalization										
**Factor**	**Univariate analysis**					**Multivariate analysis**				
	**Cause-specific hazards**		**Schoenfeld residuals**	**Subdistribution hazards**		**Cause-specific hazards**		**Schoenfeld residuals**	**Subdistribution hazards**	
	**HR (95%CI)**	***p*-value**	***p*-value**	**HR (95%CI)**	***p*-value**	**HR (95%CI)**	***p*-value**	***p*-value**	**HR (95%CI)**	***p*-value**
**Admission**										
**Total orthoedema score**	0.9 (0.53–1.53)	0.69	0.77	0.86 (0.58–1.29)	0.59					
**Log NT-proBNP (pg/mL)**	0.9 (0.66–1.21)	0.48	0.79	0.87 (0.68–1.12)	0.37	0.9 (0.60–1.36)	0.63	0.78	0.89 (0.62–1.29)	0.54
**Log hsTnT (pg/mL)**	0.77 (0.5–1.19)	0.19	0.53	0.75 (0.5–1.12)	0.14	0.81 (0.48–1.37)	0.40	0.60	0.79 (0.48–1.29)	0.35
**Log GDF-15 (pg/mL)**	1.1 (0.62–1.94)	0.75	0.90	1.03 (0.71–1.48)	0.93	1.28 (0.69–2.36)	0.43	0.91	1.24 (0.76–2.04)	0.39
**Discharge**										
**Total orthoedema score**	1.71 (0.98–2.99)	0.08	0.65	1.71 (0.99–2.94)	0.08					
**Log NT-proBNP (pg/mL)**	1.14 (0.87–1.5)	0.33	0.92	1.14 (0.93–1.4)	0.33	1.23 (0.86–1.76)	0.25	0.95	1.23 (0.90–1.69)	0.20
**Log hsTnT (pg/mL)**	0.92 (0.6–1.41)	0.69	0.58	0.92 (0.63–1.35)	0.69	0.51 (0.25–1.04)	**0.03**	0.36	0.51 (0.21–1.24)	0.14
**Log GDF-15 (pg/mL)**	1.92 (1.01–3.65)	**0.04**	0.80	1.92 (1.18–3.13)	**0.04**	2.45 (1.17–5.13)	**0.02**	0.91	2.43 (1.26–4.71)	**< 0.01**
**Diff Log NT-proBNP (pg/mL)**	0.55 (0.37–0.8)	**< 0.01**	0.87	0.55 (0.36–0.84)	**0.01**	0.66 (0.44–1.00)	0.05	0.91	0.66 (0.42–1.03)	0.07
**Diff Log hsTnT (pg/mL)**	0.5 (0.22–1.14)	0.12	**0.01**	0.5 (0.14–1.76)	0.13	0.92 (0.45–1.88)	0.81	0.16	0.92 (0.41–2.03)	0.83
**Diff Log GDF-15 (pg/mL)**	0.17 (0.06–0.46)	**< 0.01**	0.99	0.17 (0.08–0.36)	**< 0.01**	0.25 (0.08–0.78)	**0.02**	0.97	0.25 (0.10–0.65)	**< 0.01**

**Table 10:** Survival analysis for 30-day HF rehospitalization using categorical covariates

30-day heart failure rehospitalization											
**Factor**	**Threshold**	**Univariate analysis**					**Multivariate analysis**				
		**Cause-specific hazards**		**Schoenfeld residuals**	**Subdistribution hazards**		**Cause-specific hazards**		**Schoenfeld residuals**	**Subdistribution hazards**	
		**HR (95%CI)**	***p*-value**	*p***-value**	**HR (95%CI)**	***p*-value**	**HR (95%CI)**	***p*-value**	***p*-value**	**HR (95%CI)**	***p*-value**
**Admission**											
**Grading orthoedema score**			0.89	0.13		0.82					
No congestion	0	1.00 (reference)					1.00 (reference)				
Low grade	1–2	1.15 (0.14–9.17)	0.90		1.12 (0.15–8.08)	0.91					
High grade	3–4	0.86 (0.1–7.65)	0.89		0.77 (0.1–6.15)	0.80					
**NT-proBNP**	> 4577.00 pg/mL	0.76 (0.25–2.26)	0.62	0.31	0.69 (0.23–2.01)	0.50	1.53 (0.38–6.14)	0.55	0.35	1.28 (0.26–6.28)	0.76
**hsTnT**	> 38.53 pg/mL	0.24 (0.07–0.88)	**0.02**	0.12	0.23 (0.07–0.82)	**0.01**	0.18 (0.04–0.87)	**0.03**	0.17	0.2 (0.03–1.22)	0.08
**GDF-15**	> 6221.50 pg/mL	1.4 (0.44–4.4)	0.56	0.34	1.26 (0.41–3.89)	0.69	1.76 (0.54–5.70)	0.35	0.38	1.62 (0.46–5.67)	0.45
**Discharge**											
**Grading orthoedema score**	≥ 1 (congestion present)	2.92 (0.96–8.93)	0.08	0.65	2.92 (0.99–8.63)	0.08					
**NT-proBNP**	> 4577.00 pg/mL	1.71 (0.52–5.62)	0.37	1.00	1.71 (0.53–5.52)	0.37	3.99 (0.96–16.6)	0.06	0.64	3.99 (1.02–15.7)	**0.047**
**hsTnT**	> 38.53 pg/mL	0.4 (0.11–1.51)	0.15	0.53	0.4 (0.11–1.47)	0.15	0.1 (0.02–0.47)	**< 0.01**	0.80	0.1 (0.02–0.48)	**< 0.01**
**GDF-15**	> 6221.50 pg/mL	6.3 (1.36–29.2)	**< 0.01**	0.49	6.3 (1.4–28.31)	**< 0.01**	8.22 (1.70–39.7)	**< 0.01**	0.64	8.24 (1.68–40.4)	**< 0.01**
**NT-proBNP admission:discharge**	> 1.0992464	0.2 (0.04–0.95)	**0.02**	0.64	0.2 (0.05–0.92)	**0.02**	0.1 (0.01–0.81)	**0.03**	0.40	0.1 (0.01–0.78)	**0.03**
**hsTnT admission:discharge**	> 1.13408464	0.84 (0.26–2.74)	0.77	0.66	0.84 (0.26–2.7)	0.77	0.99 (0.28–3.44)	0.98	0.77	0.99 (0.29–3.37)	0.98
**GDF-15 admission:discharge**	> 1.8079528	0.41 (0.11–1.6)	0.18	0.36	0.41 (0.11–1.59)	0.18	0.4 (0.10–1.58)	0.19	0.34	0.4 (0.10–1.57)	0.19

Similarly, higher levels of NT-proBNP above 4577.00 pg/mL were associated with increased incidence of 30-day HF readmission in multivariate analysis (_SD_HR 3.99, CI 1.02–15.70). Univariate analysis also demonstrated a significant association between decrease in NT-proBNP levels from baseline to discharge and hazard and incidence of 30-day HF readmission (_CS_HR 0.55, CI 0.37–0.80; _SD_HR 0.55, CI 0.36 -0.84). Groups with over 9.0% reduction of NT-proBNP experienced significantly lower hazards and incidence of readmission in both univariate (_CS_HR 0.20, CI 0.04–0.95; _SD_HR 0.20, CI 0.05–0.92) and multivariate analyses (_CS_HR 0.10, CI 0.01–0.81; _SD_HR 0.10, CI 0.01–0.78).

Using a threshold of 38.53 pg/mL for hsTnT, levels above this value upon admission were significantly associated with an increased hazard of 30-day HF readmission in both univariate (_CS_HR 0.24, CI 0.07–0.88) and multivariate (_CS_HR 0.18, CI 0.04–0.87) analyses. Additionally, the group with elevated hsTnT levels upon admission was also associated with an increased incidence of 30-day HF rehospitalization in univariate analysis (_SD_HR 0.23, CI 0.07–0.87). Furthermore, persistently high levels of hsTnT upon discharge, as determined by this threshold, were associated with increased hazards and incidence of 30-day HF rehospitalization in multivariate analysis (_CS_HR 0.10, CI 0.02–0.47; _SD_HR 0.10, CI 0.02–0.48).

#### 90-day all-cause mortality

For 90-day all-cause mortality (Supplementary Table S19-20), a higher GDF-15 level at admission was associated with increased hazards and incidence of death in both univariate (HR 3.58, CI 1.24–10.29) and multivariate analyses (HR 3.72, CI 1.19–11.7). Using a threshold of 6221.50 pg/mL, higher GDF-15 level also demonstrated a significant association with hazards and incidence of 90-day all-cause mortality (*p* < 0.01). The log-rank test further supports this finding (*p* = 0.01), indicating that patients with different GDF-15 levels at baseline have distinct survival distributions. However, since only one event occurred in the high GDF-15 group, the model was unable to derive a hazard ratio due to non-convergence.

#### 90-day HF rehospitalization

For 90-day HF rehospitalization (Supplementary Table S13-14), GDF-15 levels at discharge demonstrated a significant association with increased hazard and incidence of 90-day HF readmission in both univariate (_CS_HR 1.71, CI 1.04–2.84; _SD_HR 1.69, CI 1.14–2.52) and multivariate analyses (_CS_HR 2.10, CI 1.15–3.82; _SD_HR 2.06, CI 1.20–3.56). Similarly, using a threshold of 6221.50 pg/mL, GDF-15 levels above this value were associated with an increased hazard and incidence of 90-day HF rehospitalization in univariate analysis (_CS_HR 2.71, CI 1.002–7.34; _SD_HR 2.68, CI 1.02–7.03).

Conversely, a reduction in GDF-15 levels from baseline to discharge showed a significant association with decreased hazard and incidence of readmission in both univariate (_CS_HR 0.2, CI 0.08–0.48; _SD_HR 0.20, CI 0.09–0.45) and multivariate analyses (_CS_HR 0.24, CI 0.09–0.61; _SD_HR 0.24, CI 0.11–0.53). Groups with over a 44.7% reduction from admission to discharge (admission:discharge ratio > 1.8079528) demonstrated a similar trend in both univariate (_CS_HR 0.30, CI 0.10–0.94; _SD_HR 0.31, CI 0.10–0.96) and multivariate analyses (_CS_HR 0.29, CI 0.09–0.90; _SD_HR 0.29, CI 0.09–0.93).

Similarly, a reduction in NT-proBNP levels from baseline to discharge showed a significant association with decreased hazard and incidence of 90-day HF readmission in univariate analysis (_CS_HR 0.65, CI 0.47–0.91; _SD_HR 0.65, CI 0.44 -0.96). Groups with over an 11.83% reduction from admission to discharge (admission:discharge ratio > 1.13408464) demonstrated a similar trend in univariate (_CS_HR 0.27, CI 0.09–0.83; _SD_HR 0.27, CI 0.09–0.80) and multivariate analyses (_CS_HR 0.19, CI 0.06–0.69; _SD_HR 0.19, CI 0.06–0.66).

Remarkably, high hsTnT levels at discharge was associated with decreased hazards of HF rehospitalization within the 90-day period from multivariate analysis (_CS_HR 0.59, CI 0.34–0.99). This relationship which only emerged from multivariate analysis may suggest potential interactions between biomarker covariates within the hazards model.

#### 180-day all-cause mortality

No association was found between biomarkers and hazards or incidence of death in 180-day all-cause mortality (Supplementary Table S21-22).

#### 180-day HF rehospitalization

For 180-day HF rehospitalization (Supplementary Table S15-16), GDF-15 reduction was significantly associated with decreased hazards and incidence in both univariate (_CS_HR 0.20 CI 0.09–0.46; _SD_HR 0.21, CI 0.10–0.44) and multivariate analyses (_CS_HR 0.23 CI 0.10–0.53; _SD_HR 0.23, CI 0.11–0.47). Accordingly, the group with over 44.7% reduction from admission to baseline was significantly associated with decreased hazards and incidence of HF readmission within the 180-day period in both univariate (_CS_HR 0.29 CI 0.10–0.79; _SD_HR 0.29, CI 0.10–0.80) and multivariate analyses (_CS_HR 0.27 CI 0.10–0.27; _SD_HR 0.27, CI 0.09–0.78).

Higher NT-proBNP levels at discharge above 4577.00 pg/mL was associated with increased hazards and incidence of 180-day HF rehospitalization in univariate analysis (_CS_HR 2.43 CI 1.01–5.87; _SD_HR 2.48, CI 1.03–5.95). Similarly, multivariate analysis also revealed significant association with increased hazards (_CS_HR 3.80 CI 1.14–12.60). In contrast, a reduction of NT-proBNP over 11.83% from baseline to discharge was associated with reduced hazards and incidence of rehospitalization in multivariate analysis (_CS_HR 0.35 CI 0.13–0.90; _SD_HR 0.34, CI 0.13–0.85).

#### All-cause mortality

For all-cause mortality over the entire follow-up period (Supplementary Table S23-24), higher NT-proBNP level at baseline was associated with increased hazard and incidence of death in univariate analysis (HR 1.33, CI 1.01–1.76). Similarly, the group with NT-proBNP levels above 4577.00 pg/mL upon admission was also significantly associated with increased hazards and incidence in both univariate (HR 3.86, CI 1.30–11.49) and multivariate analyses (HR 6.15, CI 1.71–22.10). This finding was further supported by the log-rank test which yielded significant result for NT-proBNP level at admission (*p* < 0.01), suggesting that patients with different NT-proBNP levels at baseline have distinct survival distributions.

#### HF rehospitalization

For HF rehospitalization over the entire follow-up period (Supplementary Table S17-18), reduction in GDF-15 levels from baseline to discharge was associated with decreased hazards and incidence of death in both univariate (_CS_HR 0.18, CI 0.08–0.41; _SD_HR 0.19, CI 0.09–0.45) and multivariate analyses (_CS_HR 0.20, CI 0.09–0.45; _SD_HR 0.20, CI 0.10–0.39). Similarly, the group with a reduction magnitude of over 44.7% was showed similar trend in both univariate (_CS_HR 0.26, CI 0.09–0.71; _SD_HR 0.26, CI 0.10–0.68) and multivariate analyses (_CS_HR 0.25, CI 0.09–0.68; _SD_HR 0.25, CI 0.09–0.72). In contrast, higher GDF-15 levels were with increased hazards of readmission in multivariate analysis (_CS_HR 1.71, CI 1.01–2.91).

Similarly, NT-proBNP levels above 4577.00 pg/mL were associated with increased hazards of rehospitalization over entire follow-up period in multivariate analysis (_CS_HR 3.44, CI 1.07–11.0). Accordingly, NT-proBNP reduction of over 9.0% was associated with decreased incidence of readmission (_SD_HR 0.40, CI 0.17–0.95).

## Discussion

This prospective single-center study conducted at KCMH, focusing on patients admitted for AHF syndrome regardless of ejection fraction, revealed that lower levels of GDF-15 and NT-proBNP served as valuable biomarkers for assessing the risk of HF readmission and all-cause mortality. Generally, lower levels observed upon discharge as well as a greater degree of reduction from admission to discharge were associated with a more favorable prognosis for HF readmission, while lower levels upon admission were associated with better prognosis of all-cause mortality.

Notably, a significant finding of this study was the strong correlation between reductions in GDF-15 levels and rehospitalization outcomes across all defined time points. Additionally, elevated GDF-15 levels at discharge were linked to increased hazards and incidence of early rehospitalization within both 30- and 90-day periods. Similarly, reductions in NT-proBNP were also associated with hazards and incidence of early readmission within 30 and 90 days post-discharge. These associations, which appear to be time-specific, may be attributed to non-proportional hazards observed for both GDF-15 levels at discharge and NT-proBNP reduction. However, the non-proportional hazards assumption was upheld for GDF-15 reduction, consequently resulting in the relationship being detected across all time points.

With regards to all-cause mortality, higher GDF-15 levels upon admission correlated well with increased hazards and incidence of early occurrences of death within both 30- and 90-day periods. Interestingly, higher NT-proBNP levels correlated well with increased hazards and incidence but only at a much later time when the entire follow-up period of the study was considered. Accordingly, the non-proportional hazards assumption did not hold for both of these covariates under the outcomes of all-cause mortality.

Another intriguing finding from this study is the observed association between higher levels of hsTnT, either upon admission or discharge, and a decrease in hazards and incidence of 30-day HF readmission. This finding may seem counterintuitive, as previous literature has suggested that elevated hsTnT levels are associated with an increased risk of death or HF rehospitalization. However, this discrepancy could be attributed to a limitation of this study—the patient's non-HF hospitalization status was not taken into account as a competing risk for HF readmission. Indeed, if a patient was readmitted for reasons other than HF, this would preclude HF readmission during the course of that admission but would not prevent future HF readmissions once the patient was discharged. Further data collection and analysis may be necessary to elucidate whether hsTnT increases the risk of HF readmission when considering such factors.

Finally, our study also revealed a significant association between higher orthoedema congestion score upon admission and increased hazards and incidence of all-cause mortality at 30 days. Previous clinical trials have employed an orthoedema congestion score based on the presence of orthopnea and peripheral edema. A post hoc analysis of the DOSE-HF and CARRESS-HF trials of patients with AHF with congestion (and cardiorenal syndrome in the case of CARRESS-HF) found that baseline orthoedema was moderate in 22% of patients and severe in 62% [7]. After aggressive inpatient therapy aimed at decongestion, more than a third of the patients (35%) had persistent moderate to severe congestion at discharge. Higher orthoedema scores at admission and discharge were associated with an increased risk of death at 60 days or hospitalization for HF.

GDF-15, a distant member of the transforming growth factor-β- superfamily [11], plays a crucial role in maintaining tissue homeostasis and adaptation.. During ischemia and reperfusion injury, its expression significantly increases through phosphoinositide 3-OH kinase (PI3K) and Akt-dependent signaling pathways. GDF-15 is known to protected cardiomyocytes from apoptosis as demonstrated by previous research [12]. Therefore, as our study revealed, the levels of GDF-15 can serve as predictive markers for the degree of inflammatory response and the risk of cardiovascular events.

Experimental studies suggest that various forms of cardiac stress, including pressure overload, increase the concentration of GDF-15. Animal studies have demonstrated that GDF-15 is exerts protective effects against cardiac injury due to its anti-hypertrophic [13], anti-inflammatory and anti-apoptotic properties [14]. Notably, GDF-15 is not only produced by cardiomyocytes but also by vascular smooth muscle cells, pulmonary epithelial cells, macrophages, and adipocytes in response to oxidative stress and proinflammatory signaling molecules [15].

However, clinical studies in humans indicate that higher concentrations of GDF-15 was associated with increased mortality. For example, studies by Lok et al. and Kempf et al. have shown that GDF-15 serves as a marker of marker of increased mortality in CHF [16, 17]. Lok et al. further observed that GDF-15 is an even stronger predictor than NT-proBNP [16]. Furthermore, patients with HF with reduced ejection fraction have been found to have higher levels of GDF-15 compared to those with HF with a mid-range ejection fraction [18]. Therefore, GDF-15 seems to exhibit a range functions, providing protection in certain situations while simultaneously being associated with poor outcomes in others.

Ina previous study [18], receiver operating characteristic (ROC) curves were used to estimate the associations between GDF-15 and clinical indicators in cardiac remodeling. The study found that the combination of GDF-15 and NT-proBNP (AUC = 0.905, 95%CI: 0.868–0.942, *P* < 0.001) outperformed NT-proBNP alone (AUC = 0.869, 95%CI: 0.825–0.913, *P* < 0.001) in HF. These findings suggest that incorporating GDF-15 into diagnostic assessments significantly improves the accuracy of the HF diagnosis. Plasma levels of GDF-15 indirectly reflect the degree of cardiac remodeling and fibrosis. Therefore, this study supports the use of GDF-15 as an additional prognostic factor alongside NT-proBNP.

According to a systematic review [17], there is substantial evidence indicating that GDF-15 is an independent predictor of all-cause mortality in HF. GDF-15 may provide additional predictive value for assessing the risk of HF and death in patients with MI. A multi-biomarker strategy with GDF-15 as one of the components may be superior to conventional risk scores, particularly in systemic conditions such as HF. Despite these findings, the precise role of GDF-15 in the pathophysiology of HF remains to be elucidated on a biological level. Additionally, there is a need for further research to explore how the available data on GDF-15 can be translated into therapeutic decisions regarding HF management.

In a recent meta-analysis [19] involving 6,244 patients with chronic HF, elevated circulating GDF-15 concentration is associated with a 6% increase in the risk of all-cause mortality with an increase per 1LnU in baseline GDF-15 concentration,with pooled risk ratios 1.06 (95% CI:1.03–1.10, *P* < 0.001) among chronic HF patients, especially among those with ischemic etiology, such as coronary atherosclerosis. Furthermore, the association was strengthened after excluding patients with a nonreduced ejection fraction, suggesting that GDF-15 could have a higher prognostic value in individuals with a reduced ejection fraction. Subgroup analyzes indicated that factors such as age, NYHA class and duration of follow-up may not affect the relationship between GDF-15 and the risk of all-cause death in chronic HF.

While our study was designed as a prospective analytical investigation, it still bears certain limitations. Firstly, it is important to acknowledge that this study was conducted at a single center and with a relatively small sample size. Therefore, further research may be necessary to bolster and validate our findings. Secondly, our patient cohort comprised only individuals admitted with AHF and excludes those who sought treatment through emergency room visits or outpatient clinics. This may introduce a selection bias and limit the generalizability of our results to the broader HF population. Lastly, the limited number of death cases observed in our study prevented us from identifying independent factors associated with this outcome effectively. Nevertheless, it's worth noting that these patients were enrolled over a relatively short timeframe, providing insights into the acute HF population in real-world settings.

As a result, future research should adopt a larger-scale approach, recruiting patients who received care through any channels offered by the hospital to yield more precise and comprehensive results. Additionally, our multivariate model was constrained by the inclusion of only a few covariates per analysis set due to the risk of model convergence failure when dealing with a limited dataset. Furthermore, our analysis primarily focused on linear, monotonic relationships between biomarkers and outcomes. Future studies may benefit from exploring more sophisticated models capable of incorporating a broader range of covariates and capturing potential non-linear relationships between these factors and outcomes.

Our study identified a significant connection between GDF-15 levels and numerous outcomes in patients with HF, including all-cause mortality and HF rehospitalization. These encouraging findings may offer an additional foundation for further research on this biomarker. Furthermore, the findings of this study may encourage the use of GDF-15 as an additional predictive marker in patients with HF in current clinical practices.

## Conclusion

In this single-center study, we observed a high rate of mortality and rehospitalization among patients discharged after acute HF hospitalization. Notably, we observed a significant decrease in Growth Differentiation Factor-15 (GDF-15) levels during hospitalization, suggesting its potential as a dynamic marker reflecting the course of AHF. Indeed, our findings indicated that a reduction in GDF-15 levels from baseline to discharge was associated with a decreased risk of HF rehospitalization at any time point. Lower discharge GDF-15 levels, as well as reductions in NT-proBNP levels from baseline to discharge, were also associated with a reduced risk of early HF rehospitalization at both the 30- and 90-day periods. Furthermore, higher GDF-15 levels at admission were associated with an increased risk of early all-cause mortality at 30 and 90 days, while higher NT-proBNP levels upon admission reflected a higher risk of long-term mortality. These findings underscore the potential of GDF-15 as a prognostic marker and highlight its role in predicting AHF outcomes by offering valuable insights that could inform risk stratification and personalized management strategies for AHF patients. Nevertheless, further research is warranted to validate and explore the therapeutic implications of GDF-15 in AHF.

### Supplementary Information

**Supplementary Material 1.**

## Data Availability

The datasets used and/or analyzed during the current study are available from the corresponding author upon reasonable request.
