# Supplementary material for: GDF-15: a novel biomarker of heart failure predicts short-term and long-term heart-failure rehospitalization and short-term mortality in patients with acute heart failure syndrome
Source: BMC Cardiovasc Disord. 2024 Mar 12;24:151. doi: 10.1186/s12872-024-03802-5 (PMC10936070; doi:10.1186/s12872-024-03802-5)
Supplement: Supplementary file 1 — Supplementary Material 1. [file 12872_2024_3802_MOESM1_ESM.docx]

**Supplementary Table S1** Orthoedemascore and continuous biomarker covariates in patients with 90-day HF rehospitalization

| **90-day heart failure rehospitalization** | | | | |
| --- | --- | --- | --- | --- |
| **Admission** | | | | |
| **Factors** | **Total** | **Non rehospitalization** | **Rehospitalization** | **P value** |
|  | **(N=84)** | **(N=65)** | **(N=19)** |  |
| **Total orthoedema score** |  |  |  | 0.73 |
| mean±S.D. | 2.29±0.98 | 2.26±1.00 | 2.37±0.90 |  |
| median (IQR) | 2.00 (2.00-3.00) | 2.00 (2.00-3.00) | 2.00 (2.00-3.00) |  |
| **NT-proBNP (pg/mL)** |  |  |  | 0.72 |
| mean±S.D. | 9,901±10,132 | 10,552±10,792 | 7,674±7,248 |  |
| median (IQR) | 5,529 (2,260-13,918) | 5,520 (2,065-16,544) | 5,628 (2,677-10,324) |  |
| **hsTnT (pg/mL)** |  |  |  | 0.84 |
| mean±S.D. | 217±852 | 259±964 | 75±106 |  |
| median (IQR) | 40 (27-94) | 42 (26-99) | 34 (30-75) |  |
| **Factors** | **Total** | **Non rehospitalization** | **Rehospitalization** | **P value** |
|  | **(N=83)** | **(N=65)** | **(N=18)** |  |
| **GDF-15 (pg/mL)** |  |  |  | 0.90 |
| mean±S.D. | 9,119±6,011 | 9,415±6,421 | 8,048±4,183 |  |
| median (IQR) | 6,879 (4,428-12,387) | 6,879 (4,118-13,700) | 6,646 (5,266-10,768) |  |
| **Discharge** | | | | |
| **Factors** | **Total** | **Non rehospitalization** | **Rehospitalization** | **P value** |
|  | **(N=79)** | **(N=60)** | **(N=19)** |  |
| **Total orthoedema score** |  |  |  | 0.11 |
| mean±S.D. | 0.38±0.79 | 0.30±0.72 | 0.63±0.96 |  |
| median (IQR) | 0.00 (0.00-0.00) | 0.00 (0.00-0.00) | 0.00 (0.00-2.00) |  |
| **Factors** | **Total** | **Non rehospitalization** | **Rehospitalization** | **P value** |
|  | **(N=71)** | **(N=54)** | **(N=17)** |  |
| **NT-proBNP (pg/mL)** |  |  |  | 0.13 |
| mean±S.D. | 7,091±11,041 | 7,337±12,419 | 6,307±4,649 |  |
| median (IQR) | 3,306 (690-7,863) | 2,724 (657-7,335) | 5,099 (2,974-9,758) |  |
| **hsTnT (pg/mL)** |  |  |  | 0.80 |
| mean±S.D. | 92±165 | 101±182 | 64±89 |  |
| median (IQR) | 37 (22-64) | 37 (20-78) | 35 (27-60) |  |
| **GDF-15 (pg/mL)** |  |  |  | 0.040 |
| mean±S.D. | 7,107±4,788 | 6,625±4,800 | 8,637±4,547 |  |
| median (IQR) | 5,659 (3,662-8,735) | 5,245 (3,185-8,256) | 7,143 (5,378-10,872) |  |
| **NT-proBNP admission:discharge** |  |  |  | 0.009 |
| mean±S.D. | 2.88±3.84 | 3.20±4.13 | 1.86±2.56 |  |
| median (IQR) | 1.81 (1.08-3.04) | 1.97 (1.35-3.07) | 1.34 (0.69-1.59) |  |
| **hsTnT admission:discharge** |  |  |  | 0.56 |
| mean±S.D. | 1.48±1.43 | 1.37±1.29 | 1.80±1.83 |  |
| median (IQR) | 1.13 (0.96-1.34) | 1.14 (1.00-1.34) | 1.07 (0.89-1.32) |  |
| **Factors** | **Total** | **Non rehospitalization** | **Rehospitalization** | **P value** |
|  | **(N=70)** | **(N=54)** | **(N=16)** |  |
| **GDF-15 admission:discharge** |  |  |  | 0.004 |
| mean±S.D. | 1.39±0.93 | 1.51±1.01 | 0.98±0.46 |  |
| median (IQR) | 1.10 (0.96-1.63) | 1.23 (1.00-1.69) | 0.98 (0.79-1.05) |  |

P value by Wilcoxon rank sum test, Welch Two Sample t-test

**Supplementary Table S2** Orthoedemascore and categorical biomarker covariates in patients with 90-day HF rehospitalization

| **90-day heart failure rehospitalization** | | | | |
| --- | --- | --- | --- | --- |
| **Admission** | | | | |
| **Factors** | **Total** | **Non rehospitalization** | **Rehospitalization** | **P value** |
|  | **(N=84)** | **(N=65)** | **(N=19)** |  |
| **Grading orthoedema score** |  |  |  | >0.99 |
| No congestion (0) | 6 (7.1%) | 5 (7.7%) | 1 (5.3%) |  |
| Low grade (1-2) | 47 (56%) | 36 (55%) | 11 (58%) |  |
| High grade (3-4) | 31 (37%) | 24 (37%) | 7 (37%) |  |
| **NT-proBNP (pg/mL)** |  |  |  | >0.99 |
| Low (≤4577.00) | 37 (44%) | 29 (45%) | 8 (42%) |  |
| High (>4577.00) | 47 (56%) | 36 (55%) | 11 (58%) |  |
| **hsTnT (pg/mL)** |  |  |  | 0.79 |
| Low (≤38.53) | 40 (48%) | 30 (46%) | 10 (53%) |  |
| High (>38.53) | 44 (52%) | 35 (54%) | 9 (47%) |  |
| **Factors** | **Total** | **Non rehospitalization** | **Rehospitalization** | **P value** |
|  | **(N=83)** | **(N=65)** | **(N=18)** |  |
| **GDF-15 (pg/mL)** |  |  |  | 0.79 |
| Low (≤6221.50) | 38 (46%) | 29 (45%) | 9 (50%) |  |
| High (>6221.50) | 45 (54%) | 36 (55%) | 9 (50%) |  |
| **Discharge** | | | | |
| **Factors** | **Total** | **Non rehospitalization** | **Rehospitalization** | **P value** |
|  | **(N=79)** | **(N=60)** | **(N=19)** |  |
| **Grading orthoedema score** |  |  |  | 0.18 |
| No congestion (0) | 64 (81%) | 51 (85%) | 13 (68%) |  |
| Congestion present (≥1) | 15 (19%) | 9 (15%) | 6 (32%) |  |
| **Factors** | **Total** | **Non rehospitalization** | **Rehospitalization** | **P value** |
|  | **(N=71)** | **(N=54)** | **(N=17)** |  |
| **NT-proBNP (pg/mL)** |  |  |  | 0.16 |
| Low (≤4577.00) | 41 (58%) | 34 (63%) | 7 (41%) |  |
| High (>4577.00) | 30 (42%) | 20 (37%) | 10 (59%) |  |
| **hsTnT (pg/mL)** |  |  |  | >0.99 |
| Low (≤38.53) | 38 (54%) | 29 (54%) | 9 (53%) |  |
| High (>38.53) | 33 (46%) | 25 (46%) | 8 (47%) |  |
| **GDF-15 (pg/mL)** |  |  |  | 0.093 |
| Low (≤6221.50) | 39 (55%) | 33 (61%) | 6 (35%) |  |
| High (>6221.50) | 32 (45%) | 21 (39%) | 11 (65%) |  |
| **NT-proBNP admission:discharge** |  |  |  | 0.025 |
| Low (≤1.0992464) | 36 (51%) | 23 (43%) | 13 (76%) |  |
| High (>1.0992464) | 35 (49%) | 31 (57%) | 4 (24%) |  |
| **hsTnT admission:discharge** |  |  |  | >0.99 |
| Low (≤1.13408464) | 36 (51%) | 27 (50%) | 9 (53%) |  |
| High (>1.13408464) | 35 (49%) | 27 (50%) | 8 (47%) |  |
| **Factors** | **Total** | **Non rehospitalization** | **Rehospitalization** | **P value** |
|  | **(N=70)** | **(N=54)** | **(N=16)** |  |
| **GDF-15 admission:discharge** |  |  |  | 0.044 |
| Low (≤1.8079528) | 35 (50%) | 23 (43%) | 12 (75%) |  |
| High (>1.8079528) | 35 (50%) | 31 (57%) | 4 (25%) |  |

P value by Fisher's exact test

**Supplementary Table S3** Orthoedemascore and continuous biomarker in patients with 180-day HF rehospitalization

| **180-day heart failure rehospitalization** | | | | |
| --- | --- | --- | --- | --- |
| **Admission** | | | | |
| **Factors** | **Total** | **Non rehospitalization** | **Rehospitalization** | **P value** |
|  | **(N=84)** | **(N=60)** | **(N=24)** |  |
| **Total orthoedema score** |  |  |  | 0.65 |
| mean±S.D. | 2.29±0.98 | 2.25±1.04 | 2.38±0.82 |  |
| median (IQR) | 2.00 (2.00-3.00) | 2.00 (2.00-3.00) | 2.00 (2.00-3.00) |  |
| **NT-proBNP (pg/mL)** |  |  |  | 0.63 |
| mean±S.D. | 9,901±10,132 | 9,951±10,726 | 9,777±8,681 |  |
| median (IQR) | 5,529 (2,260-13,918) | 5,109 (2,056-13,918) | 7,543 (2,679-12,962) |  |
| **hsTnT (pg/mL)** |  |  |  | 0.90 |
| mean±S.D. | 217±852 | 263±1,000 | 103±177 |  |
| median (IQR) | 40 (27-94) | 41 (26-97) | 38 (29-84) |  |
| **Factors** | **Total** | **Non rehospitalization** | **Rehospitalization** | **P value** |
|  | **(N=83)** | **(N=60)** | **(N=23)** |  |
| **GDF-15 (pg/mL)** |  |  |  | 0.66 |
| mean±S.D. | 9,119±6,011 | 9,574±6,511 | 7,930±4,352 |  |
| median (IQR) | 6,879 (4,428-12,387) | 7,146 (4,105-13,849) | 5,974 (4,778-10,595) |  |
| **Discharge** | | | | |
| **Factors** | **Total** | **Non rehospitalization** | **Rehospitalization** | **P value** |
|  | **(N=79)** | **(N=55)** | **(N=24)** |  |
| **Total orthoedema score** |  |  |  | 0.38 |
| mean±S.D. | 0.38±0.79 | 0.33±0.75 | 0.50±0.88 |  |
| median (IQR) | 0.00 (0.00-0.00) | 0.00 (0.00-0.00) | 0.00 (0.00-0.50) |  |
| **Factors** | **Total** | **Non rehospitalization** | **Rehospitalization** | **P value** |
|  | **(N=71)** | **(N=50)** | **(N=21)** |  |
| **NT-proBNP (pg/mL)** |  |  |  | 0.058 |
| mean±S.D. | 7,091±11,041 | 6,909±12,268 | 7,522±7,593 |  |
| median (IQR) | 3,306 (690-7,863) | 2,532 (657-6,449) | 6,108 (2,974-9,758) |  |
| **hsTnT (pg/mL)** |  |  |  | 0.52 |
| mean±S.D. | 92±165 | 94±175 | 88±141 |  |
| median (IQR) | 37 (22-64) | 36 (20-64) | 39 (27-63) |  |
| **GDF-15 (pg/mL)** |  |  |  | 0.10 |
| mean±S.D. | 7,107±4,788 | 6,724±4,938 | 8,020±4,386 |  |
| median (IQR) | 5,659 (3,662-8,735) | 5,254 (3,185-8,256) | 6,728 (4,497-10,582) |  |
| **NT-proBNP admission:discharge** |  |  |  | 0.042 |
| mean±S.D. | 2.88±3.84 | 3.20±4.26 | 2.12±2.52 |  |
| median (IQR) | 1.81 (1.08-3.04) | 1.96 (1.35-3.07) | 1.44 (0.69-2.48) |  |
| **hsTnT admission:discharge** |  |  |  | 0.60 |
| mean±S.D. | 1.48±1.43 | 1.39±1.33 | 1.67±1.66 |  |
| median (IQR) | 1.13 (0.96-1.34) | 1.14 (1.00-1.33) | 1.07 (0.89-1.35) |  |
| **Factors** | **Total** | **Non rehospitalization** | **Rehospitalization** | **P value** |
|  | **(N=70)** | **(N=50)** | **(N=20)** |  |
| **GDF-15 admission:discharge** |  |  |  | 0.001 |
| mean±S.D. | 1.39±0.93 | 1.55±1.03 | 0.99±0.41 |  |
| median (IQR) | 1.10 (0.96-1.63) | 1.26 (1.01-1.75) | 0.98 (0.81-1.05) |  |

P value by Wilcoxon rank sum test, Welch Two Sample t-test

**Supplementary Table S4** Orthoedemascore and categorical biomarker covariates in patients with 180-day HF rehospitalization

| **180-day heart failure rehospitalization** | | | | |
| --- | --- | --- | --- | --- |
| **Admission** | | | | |
| **Factors** | **Total** | **Non rehospitalization** | **Rehospitalization** | **P value** |
|  | **(N=84)** | **(N=60)** | **(N=24)** |  |
| **Grading orthoedema score** |  |  |  | 0.93 |
| No congestion (0) | 6 (7.1%) | 5 (8.3%) | 1 (4.2%) |  |
| Low grade (1-2) | 47 (56%) | 33 (55%) | 14 (58%) |  |
| High grade (3-4) | 31 (37%) | 22 (37%) | 9 (38%) |  |
| **NT-proBNP (pg/mL)** |  |  |  | 0.48 |
| Low (≤4577.00) | 37 (44%) | 28 (47%) | 9 (38%) |  |
| High (>4577.00) | 47 (56%) | 32 (53%) | 15 (63%) |  |
| **hsTnT (pg/mL)** |  |  |  | 0.81 |
| Low (≤38.53) | 40 (48%) | 28 (47%) | 12 (50%) |  |
| High (>38.53) | 44 (52%) | 32 (53%) | 12 (50%) |  |
| **Factors** | **Total** | **Non rehospitalization** | **Rehospitalization** | **P value** |
|  | **(N=83)** | **(N=60)** | **(N=23)** |  |
| **GDF-15 (pg/mL)** |  |  |  | 0.62 |
| Low (≤6221.50) | 38 (46%) | 26 (43%) | 12 (52%) |  |
| High (>6221.50) | 45 (54%) | 34 (57%) | 11 (48%) |  |
| **Discharge** | | | | |
| **Factors** | **Total** | **Non rehospitalization** | **Rehospitalization** | **P value** |
|  | **(N=79)** | **(N=55)** | **(N=24)** |  |
| **Grading orthoedema score** |  |  |  | 0.37 |
| No congestion (0) | 64 (81%) | 46 (84%) | 18 (75%) |  |
| Congestion present (≥1) | 15 (19%) | 9 (16%) | 6 (25%) |  |
| **Factors** | **Total** | **Non rehospitalization** | **Rehospitalization** | **P value** |
|  | **(N=71)** | **(N=50)** | **(N=21)** |  |
| **NT-proBNP (pg/mL)** |  |  |  | 0.038 |
| Low (≤4577.00) | 41 (58%) | 33 (66%) | 8 (38%) |  |
| High (>4577.00) | 30 (42%) | 17 (34%) | 13 (62%) |  |
| **hsTnT (pg/mL)** |  |  |  | 0.61 |
| Low (≤38.53) | 38 (54%) | 28 (56%) | 10 (48%) |  |
| High (>38.53) | 33 (46%) | 22 (44%) | 11 (52%) |  |
| **GDF-15 (pg/mL)** |  |  |  | 0.20 |
| Low (≤6221.50) | 39 (55%) | 30 (60%) | 9 (43%) |  |
| High (>6221.50) | 32 (45%) | 20 (40%) | 12 (57%) |  |
| **NT-proBNP admission:discharge** |  |  |  | 0.12 |
| Low (≤1.0992464) | 36 (51%) | 22 (44%) | 14 (67%) |  |
| High (>1.0992464) | 35 (49%) | 28 (56%) | 7 (33%) |  |
| **hsTnT admission:discharge** |  |  |  | >0.99 |
| Low (≤1.13408464) | 36 (51%) | 25 (50%) | 11 (52%) |  |
| High (>1.13408464) | 35 (49%) | 25 (50%) | 10 (48%) |  |
| **Factors** | **Total** | **Non rehospitalization** | **Rehospitalization** | **P value** |
|  | **(N=70)** | **(N=50)** | **(N=20)** |  |
| **GDF-15 admission:discharge** |  |  |  | 0.016 |
| Low (≤1.8079528) | 35 (50%) | 20 (40%) | 15 (75%) |  |
| High (>1.8079528) | 35 (50%) | 30 (60%) | 5 (25%) |  |

P value by Fisher's exact test

**Supplementary Table S5** Orthoedemascore and continuous biomarker covariates in patients with HF rehospitalization

| **Heart failure rehospitalization** | | | | |
| --- | --- | --- | --- | --- |
| **Admission** | | | | |
| **Factors** | **Total** | **Non rehospitalization** | **Rehospitalization** | **P value** |
|  | **(N=84)** | **(N=59)** | **(N=25)** |  |
| **Total orthoedema score** |  |  |  | 0.73 |
| mean±S.D. | 2.29±0.98 | 2.25±1.04 | 2.36±0.81 |  |
| median (IQR) | 2.00 (2.00-3.00) | 2.00 (2.00-3.00) | 2.00 (2.00-3.00) |  |
| **NT-proBNP (pg/mL)** |  |  |  | 0.70 |
| mean±S.D. | 9,901±10,132 | 10,058±10,785 | 9,531±8,587 |  |
| median (IQR) | 5,529 (2,260-13,918) | 5,409 (2,047-14,099) | 6,543 (2,682-11,768) |  |
| **hsTnT (pg/mL)** |  |  |  | 0.83 |
| mean±S.D. | 217±852 | 267±1,008 | 99±174 |  |
| median (IQR) | 40 (27-94) | 42 (27-98) | 34 (29-84) |  |
| **Factors** | **Total** | **Non rehospitalization** | **Rehospitalization** | **P value** |
|  | **(N=83)** | **(N=59)** | **(N=24)** |  |
| **GDF-15 (pg/mL)** |  |  |  | 0.44 |
| mean±S.D. | 9,119±6,011 | 9,681±6,513 | 7,736±4,361 |  |
| median (IQR) | 6,879 (4,428-12,387) | 7,413 (4,132-13,999) | 5,793 (4,692-10,423) |  |
| **Discharge** | | | | |
| **Factors** | **Total** | **Non rehospitalization** | **Rehospitalization** | **P value** |
|  | **(N=79)** | **(N=54)** | **(N=25)** |  |
| **Total orthoedema score** |  |  |  | 0.17 |
| mean±S.D. | 0.38±0.79 | 0.30±0.72 | 0.56±0.92 |  |
| median (IQR) | 0.00 (0.00-0.00) | 0.00 (0.00-0.00) | 0.00 (0.00-2.00) |  |
| **Factors** | **Total** | **Non rehospitalization** | **Rehospitalization** | **P value** |
|  | **(N=71)** | **(N=49)** | **(N=22)** |  |
| **NT-proBNP (pg/mL)** |  |  |  | 0.13 |
| mean±S.D. | 7,091±11,041 | 7,044±12,358 | 7,195±7,567 |  |
| median (IQR) | 3,306 (690-7,863) | 2,637 (679-6,509) | 5,604 (2,665-9,496) |  |
| **hsTnT (pg/mL)** |  |  |  | 0.70 |
| mean±S.D. | 92±165 | 96±176 | 85±139 |  |
| median (IQR) | 37 (22-64) | 37 (20-65) | 37 (26-62) |  |
| **GDF-15 (pg/mL)** |  |  |  | 0.11 |
| mean±S.D. | 7,107±4,788 | 6,751±4,986 | 7,901±4,317 |  |
| median (IQR) | 5,659 (3,662-8,735) | 5,116 (3,161-8,333) | 6,691 (4,716-10,169) |  |
| **NT-proBNP admission:discharge** |  |  |  | 0.11 |
| mean±S.D. | 2.88±3.84 | 3.05±4.16 | 2.52±3.09 |  |
| median (IQR) | 1.81 (1.08-3.04) | 1.95 (1.35-3.06) | 1.47 (0.70-2.65) |  |
| **hsTnT admission:discharge** |  |  |  | 0.40 |
| mean±S.D. | 1.48±1.43 | 1.40±1.35 | 1.64±1.63 |  |
| median (IQR) | 1.13 (0.96-1.34) | 1.15 (1.00-1.34) | 1.07 (0.86-1.34) |  |
| **Factors** | **Total** | **Non rehospitalization** | **Rehospitalization** | **P value** |
|  | **(N=70)** | **(N=49)** | **(N=21)** |  |
| **GDF-15 admission:discharge** |  |  |  | <0.001 |
| mean±S.D. | 1.39±0.93 | 1.57±1.04 | 0.97±0.41 |  |
| median (IQR) | 1.10 (0.96-1.63) | 1.27 (1.02-1.77) | 0.96 (0.79-1.03) |  |

P value by Wilcoxon rank sum test, Welch Two Sample t-test

**Supplementary Table S6** Orthoedemascore and categorical biomarker covariates in patients with HF rehospitalization

| **Heart failure rehospitalization** | | | | |
| --- | --- | --- | --- | --- |
| **Admission** | | | | |
| **Factors** | **Total** | **Non rehospitalization** | **Rehospitalization** | **P value** |
|  | **(N=84)** | **(N=59)** | **(N=25)** |  |
| **Grading orthoedema score** |  |  |  | 0.87 |
| No congestion (0) | 6 (7.1%) | 5 (8.5%) | 1 (4.0%) |  |
| Low grade (1-2) | 47 (56%) | 32 (54%) | 15 (60%) |  |
| High grade (3-4) | 31 (37%) | 22 (37%) | 9 (36%) |  |
| **NT-proBNP (pg/mL)** |  |  |  | 0.81 |
| Low (≤4577.00) | 37 (44%) | 27 (46%) | 10 (40%) |  |
| High (>4577.00) | 47 (56%) | 32 (54%) | 15 (60%) |  |
| **hsTnT (pg/mL)** |  |  |  | 0.64 |
| Low (≤38.53) | 40 (48%) | 27 (46%) | 13 (52%) |  |
| High (>38.53) | 44 (52%) | 32 (54%) | 12 (48%) |  |
| **Factors** | **Total** | **Non rehospitalization** | **Rehospitalization** | **P value** |
|  | **(N=83)** | **(N=59)** | **(N=24)** |  |
| **GDF-15 (pg/mL)** |  |  |  | 0.34 |
| Low (≤6221.50) | 38 (46%) | 25 (42%) | 13 (54%) |  |
| High (>6221.50) | 45 (54%) | 34 (58%) | 11 (46%) |  |
| **Discharge** | | | | |
| **Factors** | **Total** | **Non rehospitalization** | **Rehospitalization** | **P value** |
|  | **(N=79)** | **(N=54)** | **(N=25)** |  |
| **Grading orthoedema score** |  |  |  | 0.22 |
| No congestion (0) | 64 (81%) | 46 (85%) | 18 (72%) |  |
| Congestion present (≥1) | 15 (19%) | 8 (15%) | 7 (28%) |  |
| **Factors** | **Total** | **Non rehospitalization** | **Rehospitalization** | **P value** |
|  | **(N=71)** | **(N=49)** | **(N=22)** |  |
| **NT-proBNP (pg/mL)** |  |  |  | 0.071 |
| Low (≤4577.00) | 41 (58%) | 32 (65%) | 9 (41%) |  |
| High (>4577.00) | 30 (42%) | 17 (35%) | 13 (59%) |  |
| **hsTnT (pg/mL)** |  |  |  | 0.80 |
| Low (≤38.53) | 38 (54%) | 27 (55%) | 11 (50%) |  |
| High (>38.53) | 33 (46%) | 22 (45%) | 11 (50%) |  |
| **GDF-15 (pg/mL)** |  |  |  | 0.31 |
| Low (≤6221.50) | 39 (55%) | 29 (59%) | 10 (45%) |  |
| High (>6221.50) | 32 (45%) | 20 (41%) | 12 (55%) |  |
| **NT-proBNP admission:discharge** |  |  |  | 0.20 |
| Low (≤1.0992464) | 36 (51%) | 22 (45%) | 14 (64%) |  |
| High (>1.0992464) | 35 (49%) | 27 (55%) | 8 (36%) |  |
| **hsTnT admission:discharge** |  |  |  | 0.80 |
| Low (≤1.13408464) | 36 (51%) | 24 (49%) | 12 (55%) |  |
| High (>1.13408464) | 35 (49%) | 25 (51%) | 10 (45%) |  |
| **Factors** | **Total** | **Non rehospitalization** | **Rehospitalization** | **P value** |
|  | **(N=70)** | **(N=49)** | **(N=21)** |  |
| **GDF-15 admission:discharge** |  |  |  | 0.008 |
| Low (≤1.8079528) | 35 (50%) | 19 (39%) | 16 (76%) |  |
| High (>1.8079528) | 35 (50%) | 30 (61%) | 5 (24%) |  |

P value by Fisher's exact test

**Supplementary Table S7** Orthoedemascore and continuous biomarker covariates in patients with 90-day all-cause mortality

| **90-day all cause mortality** | | | | |
| --- | --- | --- | --- | --- |
| **Admission** | | | | |
| **Factors** | **Total** | **Alive** | **Deaths** | **P value** |
|  | **(N=84)** | **(N=76)** | **(N=8)** |  |
| **Total orthoedema score** |  |  |  | 0.43 |
| mean±S.D. | 2.29±0.98 | 2.26±0.94 | 2.50±1.31 |  |
| median (IQR) | 2.00 (2.00-3.00) | 2.00 (2.00-3.00) | 2.50 (2.00-3.25) |  |
| **NT-proBNP (pg/mL)** |  |  |  | 0.26 |
| mean±S.D. | 9,901±10,132 | 9,411±9,811 | 14,553±12,593 |  |
| median (IQR) | 5,529 (2,260-13,918) | 5,465 (2,260-11,929) | 12,753 (3,942-21,970) |  |
| **hsTnT (pg/mL)** |  |  |  | 0.22 |
| mean±S.D. | 217±852 | 224±893 | 152±221 |  |
| median (IQR) | 40 (27-94) | 40 (26-86) | 45 (37-162) |  |
| **Factors** | **Total** | **Alive** | **Deaths** | **P value** |
|  | **(N=83)** | **(N=76)** | **(N=7)** |  |
| **GDF-15 (pg/mL)** |  |  |  | 0.007 |
| mean±S.D. | 9,119±6,011 | 8,622±5,941 | 14,511±3,940 |  |
| median (IQR) | 6,879 (4,428-12,387) | 6,222 (4,139-11,605) | 12,880 (11,820-17,149) |  |
| **Discharge** | | | | |
| **Factors** | **Total** | **Alive** | **Deaths** | **P value** |
|  | **(N=79)** | **(N=76)** | **(N=3)** |  |
| **Total orthoedema score** |  |  |  | 0.41 |
| mean±S.D. | 0.38±0.79 | 0.39±0.80 | 0.00±0.00 |  |
| median (IQR) | 0.00 (0.00-0.00) | 0.00 (0.00-0.00) | 0.00 (0.00-0.00) |  |
| **Factors** | **Total** | **Alive** | **Deaths** | **P value** |
|  | **(N=71)** | **(N=68)** | **(N=3)** |  |
| **NT-proBNP (pg/mL)** |  |  |  | 0.35 |
| mean±S.D. | 7,091±11,041 | 7,313±11,226 | 2,050±2,340 |  |
| median (IQR) | 3,306 (690-7,863) | 3,447 (695-8,202) | 933 (706-2,836) |  |
| **hsTnT (pg/mL)** |  |  |  | 0.99 |
| mean±S.D. | 92±165 | 95±168 | 40±17 |  |
| median (IQR) | 37 (22-64) | 37 (21-66) | 32 (30-46) |  |
| **GDF-15 (pg/mL)** |  |  |  | 0.62 |
| mean±S.D. | 7,107±4,788 | 7,059±4,789 | 8,191±5,675 |  |
| median (IQR) | 5,659 (3,662-8,735) | 5,632 (3,644-8,637) | 5,811 (4,953-10,240) |  |
| **NT-proBNP admission:discharge** |  |  |  | 0.64 |
| mean±S.D. | 2.88±3.84 | 2.92±3.92 | 2.10±0.58 |  |
| median (IQR) | 1.81 (1.08-3.04) | 1.79 (1.03-3.06) | 2.39 (1.91-2.44) |  |
| **hsTnT admission:discharge** |  |  |  | 0.31 |
| mean±S.D. | 1.48±1.43 | 1.50±1.46 | 1.02±0.25 |  |
| median (IQR) | 1.13 (0.96-1.34) | 1.14 (0.97-1.35) | 0.89 (0.88-1.10) |  |
| **Factors** | **Total** | **Alive** | **Deaths** | **P value** |
|  | **(N=70)** | **(N=68)** | **(N=2)** |  |
| **GDF-15 admission:discharge** |  |  |  | 0.85 |
| mean±S.D. | 1.39±0.93 | 1.39±0.94 | 1.30±0.78 |  |
| median (IQR) | 1.10 (0.96-1.63) | 1.10 (0.96-1.61) | 1.30 (1.02-1.57) |  |

P value by Wilcoxon rank sum test, Welch Two Sample t-test

**Supplementary Table S8** Orthoedemascore and categorical biomarker covariates in patients with 90-day all-cause mortality

| **90-day all cause mortality** | | | | |
| --- | --- | --- | --- | --- |
| **Admission** | | | | |
| **Factors** | **Total** | **Alive** | **Deaths** | **P value** |
|  | **(N=84)** | **(N=76)** | **(N=8)** |  |
| **Grading orthoedema score** |  |  |  | 0.39 |
| No congestion (0) | 6 (7.1%) | 5 (6.6%) | 1 (13%) |  |
| Low grade (1-2) | 47 (56%) | 44 (58%) | 3 (38%) |  |
| High grade (3-4) | 31 (37%) | 27 (36%) | 4 (50%) |  |
| **NT-proBNP (pg/mL)** |  |  |  | 0.46 |
| Low (≤4577.00) | 37 (44%) | 35 (46%) | 2 (25%) |  |
| High (>4577.00) | 47 (56%) | 41 (54%) | 6 (75%) |  |
| **hsTnT (pg/mL)** |  |  |  | >0.99 |
| Low (≤38.53) | 40 (48%) | 36 (47%) | 4 (50%) |  |
| High (>38.53) | 44 (52%) | 40 (53%) | 4 (50%) |  |
| **Factors** | **Total** | **Alive** | **Deaths** | **P value** |
|  | **(N=83)** | **(N=76)** | **(N=7)** |  |
| **GDF-15 (pg/mL)** |  |  |  | 0.014 |
| Low (≤6221.50) | 38 (46%) | 38 (50%) | 0 (0%) |  |
| High (>6221.50) | 45 (54%) | 38 (50%) | 7 (100%) |  |
| **Discharge** | | | | |
| **Factors** | **Total** | **Alive** | **Deaths** | **P value** |
|  | **(N=79)** | **(N=76)** | **(N=3)** |  |
| **Grading orthoedema score** |  |  |  | >0.99 |
| No congestion (0) | 64 (81%) | 61 (80%) | 3 (100%) |  |
| Congestion present (≥1) | 15 (19%) | 15 (20%) | 0 (0%) |  |
| **Factors** | **Total** | **Alive** | **Deaths** | **P value** |
|  | **(N=71)** | **(N=68)** | **(N=3)** |  |
| **NT-proBNP (pg/mL)** |  |  |  | >0.99 |
| Low (≤4577.00) | 41 (58%) | 39 (57%) | 2 (67%) |  |
| High (>4577.00) | 30 (42%) | 29 (43%) | 1 (33%) |  |
| **hsTnT (pg/mL)** |  |  |  | >0.99 |
| Low (≤38.53) | 38 (54%) | 36 (53%) | 2 (67%) |  |
| High (>38.53) | 33 (46%) | 32 (47%) | 1 (33%) |  |
| **GDF-15 (pg/mL)** |  |  |  | >0.99 |
| Low (≤6221.50) | 39 (55%) | 37 (54%) | 2 (67%) |  |
| High (>6221.50) | 32 (45%) | 31 (46%) | 1 (33%) |  |
| **NT-proBNP admission:discharge** |  |  |  | 0.61 |
| Low (≤1.0992464) | 36 (51%) | 35 (51%) | 1 (33%) |  |
| High (>1.0992464) | 35 (49%) | 33 (49%) | 2 (67%) |  |
| **hsTnT admission:discharge** |  |  |  | >0.99 |
| Low (≤1.13408464) | 36 (51%) | 34 (50%) | 2 (67%) |  |
| High (>1.13408464) | 35 (49%) | 34 (50%) | 1 (33%) |  |
| **Factors** | **Total** | **Alive** | **Deaths** | **P value** |
|  | **(N=70)** | **(N=68)** | **(N=2)** |  |
| **GDF-15 admission:discharge** |  |  |  | >0.99 |
| Low (≤1.8079528) | 35 (50%) | 34 (50%) | 1 (50%) |  |
| High (>1.8079528) | 35 (50%) | 34 (50%) | 1 (50%) |  |

P value by Fisher's exact test

**Supplementary Table S9** Orthoedemascore and continuous biomarker covariates in patients with 180-day all-cause mortality

| **180-day all cause mortality** | | | | |
| --- | --- | --- | --- | --- |
| **Admission** | | | | |
| **Factors** | **Total** | **Alive** | **Deaths** | **P value** |
|  | **(N=84)** | **(N=70)** | **(N=14)** |  |
| **Total orthoedema score** |  |  |  | 0.78 |
| mean±S.D. | 2.29±0.98 | 2.29±0.93 | 2.29±1.20 |  |
| median (IQR) | 2.00 (2.00-3.00) | 2.00 (2.00-3.00) | 2.00 (2.00-3.00) |  |
| **NT-proBNP (pg/mL)** |  |  |  | 0.24 |
| mean±S.D. | 9,901±10,132 | 9,381±9,906 | 12,499±11,219 |  |
| median (IQR) | 5,529 (2,260-13,918) | 5,034 (2,235-12,132) | 8,869 (4,845-18,156) |  |
| **hsTnT (pg/mL)** |  |  |  | 0.25 |
| mean±S.D. | 217±852 | 233±929 | 136±198 |  |
| median (IQR) | 40 (27-94) | 41 (24-91) | 40 (35-130) |  |
| **Factors** | **Total** | **Alive** | **Deaths** | **P value** |
|  | **(N=83)** | **(N=70)** | **(N=13)** |  |
| **GDF-15 (pg/mL)** |  |  |  | 0.16 |
| mean±S.D. | 9,119±6,011 | 8,856±6,116 | 10,531±5,409 |  |
| median (IQR) | 6,879 (4,428-12,387) | 6,346 (4,206-11,804) | 10,761 (5,974-12,880) |  |
| **Discharge** | | | | |
| **Factors** | **Total** | **Alive** | **Deaths** | **P value** |
|  | **(N=79)** | **(N=70)** | **(N=9)** |  |
| **Total orthoedema score** |  |  |  | 0.53 |
| mean±S.D. | 0.38±0.79 | 0.40±0.81 | 0.22±0.67 |  |
| median (IQR) | 0.00 (0.00-0.00) | 0.00 (0.00-0.00) | 0.00 (0.00-0.00) |  |
| **Factors** | **Total** | **Alive** | **Deaths** | **P value** |
|  | **(N=71)** | **(N=62)** | **(N=9)** |  |
| **NT-proBNP (pg/mL)** |  |  |  | 0.20 |
| mean±S.D. | 7,091±11,041 | 7,183±11,724 | 6,455±4,259 |  |
| median (IQR) | 3,306 (690-7,863) | 2,982 (657-7,335) | 8,159 (2,974-9,758) |  |
| **hsTnT (pg/mL)** |  |  |  | 0.51 |
| mean±S.D. | 92±165 | 94±171 | 81±119 |  |
| median (IQR) | 37 (22-64) | 36 (20-65) | 39 (28-60) |  |
| **GDF-15 (pg/mL)** |  |  |  | 0.68 |
| mean±S.D. | 7,107±4,788 | 7,109±4,930 | 7,093±3,904 |  |
| median (IQR) | 5,659 (3,662-8,735) | 5,526 (3,623-8,824) | 5,811 (4,094-8,539) |  |
| **NT-proBNP admission:discharge** |  |  |  | 0.086 |
| mean±S.D. | 2.88±3.84 | 3.09±4.06 | 1.44±1.03 |  |
| median (IQR) | 1.81 (1.08-3.04) | 1.85 (1.31-3.06) | 1.13 (0.56-2.39) |  |
| **hsTnT admission:discharge** |  |  |  | 0.74 |
| mean±S.D. | 1.48±1.43 | 1.45±1.34 | 1.66±2.05 |  |
| median (IQR) | 1.13 (0.96-1.34) | 1.13 (0.97-1.35) | 1.18 (0.89-1.31) |  |
| **Factors** | **Total** | **Alive** | **Deaths** | **P value** |
|  | **(N=70)** | **(N=62)** | **(N=8)** |  |
| **GDF-15 admission:discharge** |  |  |  | 0.43 |
| mean±S.D. | 1.39±0.93 | 1.42±0.97 | 1.15±0.54 |  |
| median (IQR) | 1.10 (0.96-1.63) | 1.10 (0.97-1.63) | 1.01 (0.86-1.64) |  |

P value by Wilcoxon rank sum test, Welch Two Sample t-test

**Supplementary Table S10** Orthoedemascore and categorical biomarker covariates in patients with 180-day all-cause mortality

| **All cause mortality** | | | | |
| --- | --- | --- | --- | --- |
| **Admission** | | | | |
| **Factors** | **Total** | **Alive** | **Deaths** | **P value** |
|  | **(N=84)** | **(N=70)** | **(N=14)** |  |
| **Grading orthoedema score** |  |  |  | 0.29 |
| No congestion (0) | 6 (7.1%) | 4 (5.7%) | 2 (14%) |  |
| Low grade (1-2) | 47 (56%) | 41 (59%) | 6 (43%) |  |
| High grade (3-4) | 31 (37%) | 25 (36%) | 6 (43%) |  |
| **NT-proBNP (pg/mL)** |  |  |  | 0.080 |
| Low (≤4577.00) | 37 (44%) | 34 (49%) | 3 (21%) |  |
| High (>4577.00) | 47 (56%) | 36 (51%) | 11 (79%) |  |
| **hsTnT (pg/mL)** |  |  |  | >0.99 |
| Low (≤38.53) | 40 (48%) | 33 (47%) | 7 (50%) |  |
| High (>38.53) | 44 (52%) | 37 (53%) | 7 (50%) |  |
| **Factors** | **Total** | **Alive** | **Deaths** | **P value** |
|  | **(N=83)** | **(N=70)** | **(N=13)** |  |
| **GDF-15 (pg/mL)** |  |  |  | 0.36 |
| Low (≤6221.50) | 38 (46%) | 34 (49%) | 4 (31%) |  |
| High (>6221.50) | 45 (54%) | 36 (51%) | 9 (69%) |  |
| **Discharge** | | | | |
| **Factors** | **Total** | **Alive** | **Deaths** | **P value** |
|  | **(N=79)** | **(N=70)** | **(N=9)** |  |
| **Grading orthoedema score** |  |  |  | >0.99 |
| No congestion (0) | 64 (81%) | 56 (80%) | 8 (89%) |  |
| Congestion present (≥1) | 15 (19%) | 14 (20%) | 1 (11%) |  |
| **Factors** | **Total** | **Alive** | **Deaths** | **P value** |
|  | **(N=71)** | **(N=62)** | **(N=9)** |  |
| **NT-proBNP (pg/mL)** |  |  |  | 0.15 |
| Low (≤4577.00) | 41 (58%) | 38 (61%) | 3 (33%) |  |
| High (>4577.00) | 30 (42%) | 24 (39%) | 6 (67%) |  |
| **hsTnT (pg/mL)** |  |  |  | 0.72 |
| Low (≤38.53) | 38 (54%) | 34 (55%) | 4 (44%) |  |
| High (>38.53) | 33 (46%) | 28 (45%) | 5 (56%) |  |
| **GDF-15 (pg/mL)** |  |  |  | >0.99 |
| Low (≤6221.50) | 39 (55%) | 34 (55%) | 5 (56%) |  |
| High (>6221.50) | 32 (45%) | 28 (45%) | 4 (44%) |  |
| **NT-proBNP admission:discharge** |  |  |  | 0.48 |
| Low (≤1.0992464) | 36 (51%) | 30 (48%) | 6 (67%) |  |
| High (>1.0992464) | 35 (49%) | 32 (52%) | 3 (33%) |  |
| **hsTnT admission:discharge** |  |  |  | 0.73 |
| Low (≤1.13408464) | 36 (51%) | 32 (52%) | 4 (44%) |  |
| High (>1.13408464) | 35 (49%) | 30 (48%) | 5 (56%) |  |
| **Factors** | **Total** | **Alive** | **Deaths** | **P value** |
|  | **(N=70)** | **(N=62)** | **(N=8)** |  |
| **GDF-15 admission:discharge** |  |  |  | 0.71 |
| Low (≤1.8079528) | 35 (50%) | 30 (48%) | 5 (63%) |  |
| High (>1.8079528) | 35 (50%) | 32 (52%) | 3 (38%) |  |

P value by Fisher's exact test

**Supplementary Table S11** Orthoedemascore and continuous biomarker covariates in patients with all-cause mortality

| **All cause mortality** | | | | |
| --- | --- | --- | --- | --- |
| **Admission** | | | | |
| **Factors** | **Total** | **Alive** | **Deaths** | **P value** |
|  | **(N=84)** | **(N=70)** | **(N=14)** |  |
| **Total orthoedema score** |  |  |  | 0.84 |
| mean±S.D. | 2.29±0.98 | 2.30±0.98 | 2.24±1.00 |  |
| median (IQR) | 2.00 (2.00-3.00) | 2.00 (2.00-3.00) | 2.00 (2.00-3.00) |  |
| **NT-proBNP (pg/mL)** |  |  |  | 0.045 |
| mean±S.D. | 9,901±10,132 | 8,712±9,560 | 13,468±11,178 |  |
| median (IQR) | 5,529 (2,260-13,918) | 4,212 (2,144-11,508) | 9,194 (4,955-22,954) |  |
| **hsTnT (pg/mL)** |  |  |  | 0.26 |
| mean±S.D. | 217±852 | 252±978 | 113±166 |  |
| median (IQR) | 40 (27-94) | 42 (22-89) | 39 (34-102) |  |
| **Factors** | **Total** | **Alive** | **Deaths** | **P value** |
|  | **(N=73)** | **(N=63)** | **(N=20)** |  |
| **GDF-15 (pg/mL)** |  |  |  | 0.15 |
| mean±S.D. | 9,119±6,011 | 8,709±6,035 | 10,411±5,895 |  |
| median (IQR) | 6,879 (4,428-12,387) | 6,224 (4,107-11,802) | 9,696 (5,526-13,234) |  |
| **Discharge** | | | | |
| **Factors** | **Total** | **Alive** | **Deaths** | **P value** |
|  | **(N=79)** | **(N=70)** | **(N=9)** |  |
| **Total orthoedema score** |  |  |  | 0.99 |
| mean±S.D. | 0.38±0.79 | 0.38±0.79 | 0.38±0.81 |  |
| median (IQR) | 0.00 (0.00-0.00) | 0.00 (0.00-0.00) | 0.00 (0.00-0.00) |  |
| **Factors** | **Total** | **Alive** | **Deaths** | **P value** |
|  | **(N=71)** | **(N=56)** | **(N=15)** |  |
| **NT-proBNP (pg/mL)** |  |  |  | 0.090 |
| mean±S.D. | 7,091±11,041 | 7,195±12,179 | 6,700±5,154 |  |
| median (IQR) | 3,306 (690-7,863) | 2,862 (632-7,294) | 6,108 (2,768-9,235) |  |
| **hsTnT (pg/mL)** |  |  |  | 0.50 |
| mean±S.D. | 92±165 | 99±179 | 68±94 |  |
| median (IQR) | 37 (22-64) | 35 (19-69) | 39 (27-58) |  |
| **GDF-15 (pg/mL)** |  |  |  | 0.78 |
| mean±S.D. | 7,107±4,788 | 7,106±4,905 | 7,111±4,480 |  |
| median (IQR) | 5,659 (3,662-8,735) | 5,526 (3,561-9,243) | 5,811 (3,931-8,366) |  |
| **NT-proBNP admission:discharge** |  |  |  | 0.32 |
| mean±S.D. | 2.88±3.84 | 3.13±4.21 | 1.98±1.76 |  |
| median (IQR) | 1.81 (1.08-3.04) | 1.85 (1.23-3.06) | 1.54 (0.96-2.44) |  |
| **hsTnT admission:discharge** |  |  |  | 0.90 |
| mean±S.D. | 1.48±1.43 | 1.43±1.35 | 1.65±1.75 |  |
| median (IQR) | 1.13 (0.96-1.34) | 1.13 (0.97-1.33) | 1.18 (0.88-1.34) |  |
| **Factors** | **Total** | **Alive** | **Deaths** | **P value** |
|  | **(N=70)** | **(N=56)** | **(N=14)** |  |
| **GDF-15 admission:discharge** |  |  |  | 0.42 |
| mean±S.D. | 1.39±0.93 | 1.42±0.99 | 1.25±0.69 |  |
| median (IQR) | 1.10 (0.96-1.63) | 1.12 (0.98-1.64) | 1.05 (0.84-1.51) |  |

P value by Wilcoxon rank sum test, Welch Two Sample t-test and Fisher's exact test

**Supplementary Table S12** Orthoedemascore and categorical biomarker covariates in patients with all-cause mortality

| **180-day all cause mortality** | | | | |
| --- | --- | --- | --- | --- |
| **Admission** | | | | |
| **Factors** | **Total** | **Alive** | **Deaths** | **P value** |
|  | **(N=84)** | **(N=63)** | **(N=21)** |  |
| **Grading orthoedema score** |  |  |  | 0.79 |
| No congestion (0) | 6 (7.1%) | 4 (6.3%) | 2 (9.5%) |  |
| Low grade (1-2) | 47 (56%) | 35 (56%) | 12 (57%) |  |
| High grade (3-4) | 31 (37%) | 24 (38%) | 7 (33%) |  |
| **NT-proBNP (pg/mL)** |  |  |  | 0.011 |
| Low (≤4577.00) | 37 (44%) | 33 (52%) | 4 (19%) |  |
| High (>4577.00) | 47 (56%) | 30 (48%) | 17 (81%) |  |
| **hsTnT (pg/mL)** |  |  |  | >0.99 |
| Low (≤38.53) | 40 (48%) | 30 (48%) | 10 (48%) |  |
| High (>38.53) | 44 (52%) | 33 (52%) | 11 (52%) |  |
| **Factors** | **Total** | **Alive** | **Deaths** | **P value** |
|  | **(N=73)** | **(N=63)** | **(N=20)** |  |
| **GDF-15 (pg/mL)** |  |  |  | 0.31 |
| Low (≤6221.50) | 38 (46%) | 31 (49%) | 7 (35%) |  |
| High (>6221.50) | 45 (54%) | 32 (51%) | 13 (65%) |  |
| **Discharge** | | | | |
| **Factors** | **Total** | **Alive** | **Deaths** | **P value** |
|  | **(N=79)** | **(N=63)** | **(N=16)** |  |
| **Grading orthoedema score** |  |  |  | >0.99 |
| No congestion (0) | 64 (81%) | 51 (81%) | 13 (81%) |  |
| Congestion present (≥1) | 15 (19%) | 12 (19%) | 3 (19%) |  |
| **Factors** | **Total** | **Alive** | **Deaths** | **P value** |
|  | **(N=71)** | **(N=56)** | **(N=15)** |  |
| **NT-proBNP (pg/mL)** |  |  |  | 0.15 |
| Low (≤4577.00) | 41 (58%) | 35 (63%) | 6 (40%) |  |
| High (>4577.00) | 30 (42%) | 21 (38%) | 9 (60%) |  |
| **hsTnT (pg/mL)** |  |  |  | 0.57 |
| Low (≤38.53) | 38 (54%) | 31 (55%) | 7 (47%) |  |
| High (>38.53) | 33 (46%) | 25 (45%) | 8 (53%) |  |
| **GDF-15 (pg/mL)** |  |  |  | 0.77 |
| Low (≤6221.50) | 39 (55%) | 30 (54%) | 9 (60%) |  |
| High (>6221.50) | 32 (45%) | 26 (46%) | 6 (40%) |  |
| **NT-proBNP admission:discharge** |  |  |  | 0.56 |
| Low (≤1.0992464) | 36 (51%) | 27 (48%) | 9 (60%) |  |
| High (>1.0992464) | 35 (49%) | 29 (52%) | 6 (40%) |  |
| **hsTnT admission:discharge** |  |  |  | 0.78 |
| Low (≤1.13408464) | 36 (51%) | 29 (52%) | 7 (47%) |  |
| High (>1.13408464) | 35 (49%) | 27 (48%) | 8 (53%) |  |
| **Factors** | **Total** | **Alive** | **Deaths** | **P value** |
|  | **(N=70)** | **(N=56)** | **(N=14)** |  |
| **GDF-15 admission:discharge** |  |  |  | 0.37 |
| Low (≤1.8079528) | 35 (50%) | 26 (46%) | 9 (64%) |  |
| High (>1.8079528) | 35 (50%) | 30 (54%) | 5 (36%) |  |

P value by Fisher's exact test

**Supplementary Table S13** Survival analysis for 90-day HF rehospitalization using continuous covariates

| **90-day heart failure rehospitalization** | | | | | | | | | | |
| --- | --- | --- | --- | --- | --- | --- | --- | --- | --- | --- |
| **Factor** | **Univariate analysis** | | | | | **Multivariate analysis** | | | | |
|  | **Cause-specific hazards** | | **Schoenfeld residuals** | **Subdistribution hazards** | | **Cause-specific hazards** | | **Schoenfeld residuals** | **Subdistribution hazards** | |
|  | **HR (95%CI)** | **p-value** | **p-value** | **HR (95%CI)** | **p-value** | **HR (95%CI)** | **p-value** | **p-value** | **HR (95%CI)** | **p-value** |
| **Admission** | | | | | | | | | | |
| **Total orthoedema score** | 1.12 (0.71-1.79) | 0.62 | 0.19 | 1.08 (0.72-1.61) | 0.75 |  |  |  |  |  |
| **Log NT-proBNP (pg/mL)** | 0.99 (0.77-1.27) | 0.92 | 0.44 | 0.96 (0.78-1.18) | 0.73 | 0.97 (0.70-1.36) | 0.88 | 0.38 | 0.96 (0.71-1.31) | 0.82 |
| **Log hsTnT (pg/mL)** | 0.93 (0.7-1.23) | 0.59 | 0.14 | 0.9 (0.72-1.13) | 0.46 | 0.94 (0.66-1.33) | 0.71 | 0.18 | 0.93 (0.70-1.23) | 0.60 |
| **Log GDF-15 (pg/mL)** | 1.07 (0.68-1.7) | 0.76 | 0.69 | 0.99 (0.71-1.38) | 0.96 | 1.13 (0.68-1.86) | 0.64 | 0.70 | 1.06 (0.68-1.65) | 0.81 |
| **Discharge** | | | | | | | | | | |
| **Total orthoedema score** | 1.5 (0.93-2.44) | 0.12 | 0.75 | 1.51 (0.94-2.43) | 0.12 |  |  |  |  |  |
| **Log NT-proBNP (pg/mL)** | 1.14 (0.92-1.41) | 0.22 | 0.89 | 1.15 (0.96-1.37) | 0.21 | 1.21 (0.91-1.61) | 0.18 | 0.86 | 1.23 (0.95-1.59) | 0.12 |
| **Log hsTnT (pg/mL)** | 0.97 (0.7-1.34) | 0.84 | 0.87 | 0.97 (0.74-1.27) | 0.84 | 0.59 (0.34-1.00) | **0.03** | 0.99 | 0.58 (0.32-1.05) | 0.07 |
| **Log GDF-15 (pg/mL)** | 1.71 (1.04-2.84) | **0.03** | 0.51 | 1.69 (1.14-2.52) | **0.04** | 2.1 (1.15-3.82) | **0.01** | 0.63 | 2.06 (1.20-3.56) | **<0.01** |
| **Diff Log NT-proBNP (pg/mL)** | 0.65 (0.47-0.91) | **0.02** | 0.29 | 0.65 (0.44-0.96) | **0.02** | 0.77 (0.56-1.06) | 0.11 | 0.35 | 0.76 (0.53-1.09) | 0.14 |
| **Diff Log hsTnT (pg/mL)** | 0.93 (0.45-1.89) | 0.83 | **<0.01** | 0.93 (0.25-3.48) | 0.84 | 1.11 (0.65-1.91) | 0.70 | 0.10 | 1.12 (0.61-2.06) | 0.71 |
| **Diff Log GDF-15 (pg/mL)** | 0.2 (0.08-0.48) | **<0.01** | 0.83 | 0.2 (0.09-0.45) | **<0.01** | 0.24 (0.09-0.61) | **<0.01** | 0.75 | 0.24 (0.11-0.53) | **<0.01** |

**Supplementary Table S14** Survival analysis for 90-day HF rehospitalization using categorical covariates

| **90-day heart failure rehospitalization** | | | | | | | | | | | |
| --- | --- | --- | --- | --- | --- | --- | --- | --- | --- | --- | --- |
| **Factor** | **Threshold** | **Univariate analysis** | | | | | **Multivariate analysis** | | | | |
|  |  | **Cause-specific hazards** | | **Schoenfeld residuals** | **Subdistribution hazards** | | **Cause-specific hazards** | | **Schoenfeld residuals** | **Subdistribution hazards** | |
|  |  | **HR (95%CI)** | **p-value** | **p-value** | **HR (95%CI)** | **p-value** | **HR (95%CI)** | **p-value** | **p-value** | **HR (95%CI)** | **p-value** |
| **Admission** | | | | | | | | | | | |
| **Grading orthoedema score** |  |  | 0.91 | 0.25 |  | 0.92 |  |  |  |  |  |
| No congestion | 0 | 1.00 (reference) |  |  |  |  | 1.00 (reference) |  |  |  |  |
| Low grade | 1-2 | 1.51 (0.2-11.72) | 0.69 |  | 1.48 (0.19-11.34) | 0.70 |  |  |  |  |  |
| High grade | 3-4 | 1.48 (0.18-12.04) | 0.71 |  | 1.35 (0.17-10.69) | 0.78 |  |  |  |  |  |
| **NT-proBNP** | >4577.00 pg/mL | 1.18 (0.48-2.94) | 0.72 | 0.45 | 1.06 (0.43-2.61) | 0.91 | 1.45 (0.43-4.90) | 0.55 | 0.34 | 1.23 (0.31-4.97) | 0.77 |
| **hsTnT** | >38.53 pg/mL | 0.72 (0.29-1.78) | 0.48 | **<0.01** | 0.71 (0.3-1.73) | 0.46 | 0.63 (0.19-2.08) | 0.45 | **<0.01** | 0.71 (0.20-2.54) | 0.60 |
| **GDF-15** | >6221.50 pg/mL | 1.02 (0.41-2.57) | 0.97 | 0.10 | 0.88 (0.35-2.18) | 0.78 | 1.01 (0.39-2.67) | 0.98 | 0.11 | 0.88 (0.29-2.67) | 0.82 |
| **Discharge** | | | | | | | | | | | |
| **Grading orthoedema score** | ≥1 (congestion present) | 2.26 (0.86-5.94) | 0.12 | 0.75 | 2.28 (0.88-5.92) | 0.12 |  |  |  |  |  |
| **NT-proBNP** | >4577.00 pg/mL | 2.08 (0.79-5.46) | 0.13 | 0.61 | 2.11 (0.81-5.48) | 0.13 | 3.44 (0.94-12.6) | 0.06 | 0.47 | 3.51 (0.81-15.2) | 0.09 |
| **hsTnT** | >38.53 pg/mL | 0.94 (0.36-2.44) | 0.90 | **0.02** | 0.94 (0.37-2.38) | 0.89 | 0.29 (0.08-1.09) | 0.07 | **0.04** | 0.29 (0.07-1.23) | 0.09 |
| **GDF-15** | >6221.50 pg/mL | 2.71 (1-7.34) | **0.04** | 0.07 | 2.68 (1.02-7.03) | **0.046** | 2.8 (0.96-8.17) | 0.06 | 0.10 | 2.81 (0.87-9.07) | 0.09 |
| **NT-proBNP admission:discharge** | >1.0992464 | 0.27 (0.09-0.83) | **0.01** | 0.54 | 0.27 (0.09-0.8) | **0.01** | 0.19 (0.06-0.69) | **0.01** | 0.31 | 0.19 (0.06-0.66) | **0.01** |
| **hsTnT admission:discharge** | >1.13408464 | 0.89 (0.34-2.32) | 0.82 | 0.86 | 0.9 (0.35-2.3) | 0.83 | 0.99 (0.37-2.66) | 0.99 | 0.86 | 1.0 (0.38-2.58) | 1.00 |
| **GDF-15 admission:discharge** | >1.8079528 | 0.3 (0.1-0.94) | **0.03** | 0.16 | 0.31 (0.1-0.96) | **0.03** | 0.29 (0.09-0.90) | **0.03** | 0.14 | 0.29 (0.09-0.93) | **0.04** |

**Supplementary Table S15** Survival analysis for 180-day HF rehospitalization using continuous covariates

| **180-day heart failure rehospitalization** | | | | | | | | | | |
| --- | --- | --- | --- | --- | --- | --- | --- | --- | --- | --- |
| **Factor** | **Univariate analysis** | | | | | **Multivariate analysis** | | | | |
|  | **Cause-specific hazards** | | **Schoenfeld residuals** | **Subdistribution hazards** | | **Cause-specific hazards** | | **Schoenfeld residuals** | **Subdistribution hazards** | |
|  | **HR (95%CI)** | **p-value** | **p-value** | **HR (95%CI)** | **p-value** | **HR (95%CI)** | **p-value** | **p-value** | **HR (95%CI)** | **p-value** |
| **Admission** | | | | | | | | | | |
| **Total orthoedema score** | 1.15 (0.76-1.74) | 0.51 | 0.24 | 1.1 (0.77-1.57) | 0.65 |  |  |  |  |  |
| **Log NT-proBNP (pg/mL)** | 1.08 (0.86-1.35) | 0.53 | 0.08 | 1.04 (0.85-1.28) | 0.72 | 1.1 (0.81-1.49) | 0.53 | 0.06 | 1.1 (0.82-1.47) | 0.54 |
| **Log hsTnT (pg/mL)** | 0.98 (0.78-1.23) | 0.88 | 0.09 | 0.96 (0.79-1.17) | 0.72 | 0.94 (0.70-1.25) | 0.67 | 0.10 | 0.93 (0.74-1.19) | 0.57 |
| **Log GDF-15 (pg/mL)** | 1.04 (0.7-1.55) | 0.85 | 0.62 | 0.95 (0.7-1.3) | 0.81 | 1.0 (0.64-1.56) | 0.98 | 0.60 | 0.92 (0.61-1.38) | 0.68 |
| **Discharge** | | | | | | | | | | |
| **Total orthoedema score** | 1.28 (0.8-2.03) | 0.32 | 0.20 | 1.29 (0.8-2.07) | 0.30 |  |  |  |  |  |
| **Log NT-proBNP (pg/mL)** | 1.15 (0.95-1.39) | 0.15 | 0.70 | 1.16 (0.97-1.38) | 0.14 | 1.18 (0.91-1.52) | 0.21 | 0.70 | 1.19 (0.94-1.51) | 0.14 |
| **Log hsTnT (pg/mL)** | 1.04 (0.8-1.36) | 0.77 | 0.33 | 1.04 (0.82-1.33) | 0.76 | 0.74 (0.48-1.13) | 0.14 | 0.31 | 0.73 (0.46-1.18) | 0.20 |
| **Log GDF-15 (pg/mL)** | 1.53 (0.98-2.39) | 0.06 | 0.25 | 1.51 (1.05-2.18) | 0.07 | 1.65 (0.96-2.82) | 0.07 | 0.28 | 1.62 (0.95-2.74) | 0.08 |
| **Diff Log NT-proBNP (pg/mL)** | 0.72 (0.53-0.99) | 0.05 | 0.11 | 0.72 (0.5-1.03) | 0.05 | 0.84 (0.64-1.11) | 0.23 | 0.21 | 0.83 (0.60-1.16) | 0.28 |
| **Diff Log hsTnT (pg/mL)** | 0.88 (0.44-1.74) | 0.70 | **0.02** | 0.88 (0.26-3.03) | 0.72 | 1.02 (0.62-1.69) | 0.93 | 0.42 | 1.04 (0.59-1.80) | 0.90 |
| **Diff Log GDF-15 (pg/mL)** | 0.2 (0.09-0.46) | **<0.01** | 0.83 | 0.21 (0.1-0.44) | **<0.01** | 0.23 (0.10-0.53) | **<0.01** | 0.84 | 0.23 (0.11-0.47) | **<0.01** |

**Supplementary Table S16** Survival analysis for 180-day HF rehospitalization using categorical variables

| **180-day heart failure rehospitalization** | | | | | | | | | | | |
| --- | --- | --- | --- | --- | --- | --- | --- | --- | --- | --- | --- |
| **Factor** | **Threshold** | **Univariate analysis** | | | | | **Multivariate analysis** | | | | |
|  |  | **Cause-specific hazards** | | **Schoenfeld residuals** | **Subdistribution hazards** | | **Cause-specific hazards** | | **Schoenfeld residuals** | **Subdistribution hazards** | |
|  |  | **HR (95%CI)** | **p-value** | **p-value** | **HR (95%CI)** | **p-value** | **HR (95%CI)** | **p-value** | **p-value** | **HR (95%CI)** | **p-value** |
| **Admission** | | | | | | | | | | | |
| **Grading orthoedema score** |  | 0 (0-0) | 0.78 | 0.33 | 0 (0-0) | 0.78 |  |  |  |  |  |
| No congestion | 0 | 1.00 (reference) |  |  |  |  | 1.00 (reference) |  |  |  |  |
| Low grade | 1-2 | 1.9 (0.25-14.45) | 0.54 |  | 1.93 (0.25-15.12) | 0.53 |  |  |  |  |  |
| High grade | 3-4 | 1.92 (0.24-15.19) | 0.53 |  | 1.8 (0.22-14.54) | 0.58 |  |  |  |  |  |
| **NT-proBNP** | >4577.00 pg/mL | 1.44 (0.63-3.29) | 0.38 | 0.18 | 1.29 (0.57-2.95) | 0.54 | 1.98 (0.67-5.81) | 0.22 | 0.12 | 1.68 (0.52-5.44) | 0.39 |
| **hsTnT** | >38.53 pg/mL | 0.81 (0.36-1.81) | 0.61 | **<0.01** | 0.8 (0.37-1.77) | 0.60 | 0.6 (0.21-1.69) | 0.33 | **0.01** | 0.7 (0.24-1.99) | 0.50 |
| **GDF-15** | >6221.50 pg/mL | 0.93 (0.41-2.11) | 0.86 | 0.14 | 0.79 (0.35-1.78) | 0.58 | 0.85 (0.36-2.01) | 0.71 | 0.13 | 0.72 (0.27-1.87) | 0.50 |
| **Discharge** | | | | | | | | | | | |
| **Grading orthoedema score** | ≥1 (congestion present) | 1.63 (0.65-4.11) | 0.32 | 0.20 | 1.66 (0.64-4.3) | 0.30 |  |  |  |  |  |
| **NT-proBNP** | >4577.00 pg/mL | 2.43 (1.01-5.87) | **0.04** | 0.32 | 2.48 (1.03-5.95) | **0.04** | 3.8 (1.14-12.6) | **0.03** | 0.26 | 3.81 (0.98-14.9) | 0.05 |
| **hsTnT** | >38.53 pg/mL | 1.19 (0.5-2.8) | 0.69 | **0.01** | 1.18 (0.51-2.76) | 0.70 | 0.39 (0.12-1.30) | 0.13 | **0.02** | 0.39 (0.10-1.52) | 0.18 |
| **GDF-15** | >6221.50 pg/mL | 2.01 (0.85-4.78) | 0.11 | **0.03** | 1.99 (0.86-4.61) | 0.116 | 1.8 (0.70-4.63) | 0.22 | **0.03** | 1.82 (0.65-5.12) | 0.26 |
| **NT-proBNP admission:discharge** | >1.0992464 | 0.43 (0.17-1.06) | 0.06 | 0.12 | 0.43 (0.18-1.03) | 0.06 | 0.35 (0.13-0.90) | **0.03** | 0.06 | 0.34 (0.13-0.85) | **0.02** |
| **hsTnT admission:discharge** | >1.13408464 | 0.91 (0.39-2.15) | 0.83 | 0.84 | 0.91 (0.39-2.12) | 0.84 | 0.98 (0.41-2.37) | 0.97 | 0.83 | 0.99 (0.41-2.36) | 0.98 |
| **GDF-15 admission:discharge** | >1.8079528 | 0.29 (0.1-0.79) | **<0.01** | 0.28 | 0.29 (0.1-0.8) | **<0.01** | 0.27 (0.10-0.75) | **0.01** | 0.24 | 0.27 (0.09-0.78) | **0.02** |

**Supplementary Table S17** Survival analysis for HF rehospitalization using continuous covariates

| **Heart failure rehospitalization** | | | | | | | | | | |
| --- | --- | --- | --- | --- | --- | --- | --- | --- | --- | --- |
| **Factor** | **Univariate analysis** | | | | | **Multivariate analysis** | | | | |
|  | **Cause-specific hazards** | | **Schoenfeld residuals** | **Subdistribution hazards** | | **Cause-specific hazards** | | **Schoenfeld residuals** | **Subdistribution hazards** | |
|  | **HR (95%CI)** | **p-value** | **p-value** | **HR (95%CI)** | **p-value** | **HR (95%CI)** | **p-value** | **p-value** | **HR (95%CI)** | **p-value** |
| **Admission** | | | | | | | | | | |
| **Total orthoedema score** | 1.14 (0.76-1.71) | 0.54 | 0.32 | 1.09 (0.77-1.54) | 0.68 |  |  |  |  |  |
| **Log NT-proBNP (pg/mL)** | 1.29 (0.58-2.88) | 0.53 | 0.43 | 1.16 (0.52-2.57) | 0.72 | 1.86 (0.65-5.34) | 0.25 | 0.30 | 1.58 (0.51-4.89) | 0.43 |
| **Log hsTnT (pg/mL)** | 0.75 (0.34-1.64) | 0.47 | **0.03** | 0.74 (0.34-1.61) | 0.46 | 0.57 (0.21-1.60) | 0.29 | 0.05 | 0.67 (0.24-1.87) | 0.44 |
| **Log GDF-15 (pg/mL)** | 0.86 (0.38-1.91) | 0.70 | 0.08 | 0.72 (0.33-1.6) | 0.43 | 0.8 (0.34-1.87) | 0.61 | 0.07 | 0.67 (0.26-1.73) | 0.41 |
| **Discharge** | | | | | | | | | | |
| **Total orthoedema score** | 1.37 (0.88-2.12) | 0.18 | 0.51 | 1.38 (0.89-2.14) | 0.17 |  |  |  |  |  |
| **Log NT-proBNP (pg/mL)** | 1.11 (0.92-1.33) | 0.28 | 0.76 | 1.11 (0.94-1.32) | 0.25 | 1.12 (0.88-1.44) | 0.36 | 0.73 | 1.14 (0.91-1.44) | 0.26 |
| **Log hsTnT (pg/mL)** | 1.02 (0.78-1.33) | 0.90 | 0.53 | 1.02 (0.8-1.3) | 0.88 | 0.74 (0.49-1.13) | 0.14 | 0.53 | 0.74 (0.46-1.19) | 0.21 |
| **Log GDF-15 (pg/mL)** | 1.51 (0.98-2.33) | 0.06 | 0.23 | 1.48 (1.03-2.12) | 0.08 | 1.71 (1.01-2.91) | **0.047** | 0.27 | 1.65 (0.98-2.78) | 0.06 |
| **Diff Log NT-proBNP (pg/mL)** | 0.79 (0.57-1.09) | 0.15 | **0.02** | 0.79 (0.55-1.13) | 0.15 | 0.91 (0.70-1.18) | 0.47 | 0.08 | 0.9 (0.68-1.19) | 0.46 |
| **Diff Log hsTnT (pg/mL)** | 0.83 (0.42-1.66) | 0.58 | 0.07 | 0.83 (0.24-2.94) | 0.60 | 0.98 (0.60-1.58) | 0.93 | 0.71 | 0.99 (0.59-1.66) | 0.98 |
| **Diff Log GDF-15 (pg/mL)** | 0.18 (0.08-0.41) | **<0.01** | 0.79 | 0.19 (0.09-0.38) | **<0.01** | 0.2 (0.09-0.45) | **<0.01** | 0.74 | 0.2 (0.10-0.39) | **<0.01** |

**Supplementary Table S18** Survival analysis for HF rehospitalization using categorical variables

| **Heart failure rehospitalization** | | | | | | | | | | | |
| --- | --- | --- | --- | --- | --- | --- | --- | --- | --- | --- | --- |
| **Factor** | **Threshold** | **Univariate analysis** | | | | | **Multivariate analysis** | | | | |
|  |  | **Cause-specific hazards** | | **Schoenfeld residuals** | **Subdistribution hazards** | | **Cause-specific hazards** | | **Schoenfeld residuals** | **Subdistribution hazards** | |
|  |  | **HR (95%CI)** | **p-value** | **p-value** | **HR (95%CI)** | **p-value** | **HR (95%CI)** | **p-value** | **p-value** | **HR (95%CI)** | **p-value** |
| **Admission** | | | | | | | | | | | |
| **Grading orthoedema score** |  | 0 (0-0) | 0.75 | 0.47 | 0 (0-0) | 0.73 |  |  |  |  |  |
| No congestion | 0 | 1.00 (reference) |  |  |  |  | 1.00 (reference) |  |  |  |  |
| Low grade | 1-2 | 2.03 (0.27-15.36) | 0.49 |  | 2.06 (0.26-16.17) | 0.49 |  |  |  |  |  |
| High grade | 3-4 | 1.94 (0.25-15.35) | 0.53 |  | 1.82 (0.22-14.84) | 0.58 |  |  |  |  |  |
| **NT-proBNP** | >4577.00 pg/mL | 1.29 (0.58-2.88) | 0.53 | 0.43 | 1.16 (0.52-2.57) | 0.72 | 1.86 (0.65-5.34) | 0.25 | 0.30 | 1.58 (0.51-4.89) | 0.43 |
| **hsTnT** | >38.53 pg/mL | 0.75 (0.34-1.64) | 0.47 | 0.03 | 0.74 (0.34-1.61) | 0.46 | 0.57 (0.21-1.60) | 0.29 | 0.05 | 0.67 (0.24-1.87) | 0.44 |
| **GDF-15** | >6221.50 pg/mL | 0.86 (0.38-1.91) | 0.70 | 0.08 | 0.72 (0.33-1.6) | 0.43 | 0.8 (0.34-1.87) | 0.61 | 0.07 | 0.67 (0.26-1.73) | 0.41 |
| **Discharge** | | | | | | | | | | | |
| **Grading orthoedema score** | ≥1 (congestion present) | 1.87 (0.78-4.47) | 0.18 | 0.51 | 1.91 (0.8-4.58) | 0.17 |  |  |  |  |  |
| **NT-proBNP** | >4577.00 pg/mL | 2.12 (0.91-4.98) | 0.08 | 0.69 | 2.18 (0.93-5.09) | 0.07 | 3.44 (1.07-11.0) | **0.04** | 0.61 | 3.45 (0.92-12.9) | 0.07 |
| **hsTnT** | >38.53 pg/mL | 1.07 (0.46-2.46) | 0.88 | 0.05 | 1.07 (0.47-2.45) | 0.87 | 0.38 (0.12-1.24) | 0.11 | 0.09 | 0.39 (0.10-1.47) | 0.16 |
| **GDF-15** | >6221.50 pg/mL | 1.82 (0.79-4.22) | 0.16 | **0.02** | 1.8 (0.8-4.08) | 0.17 | 1.7 (0.68-4.27) | 0.26 | **0.02** | 1.71 (0.62-4.68) | 0.30 |
| **NT-proBNP admission:discharge** | >1.0992464 | 0.5 (0.21-1.19) | 0.11 | **0.04** | 0.5 (0.21-1.17) | 0.11 | 0.4 (0.16-1.01) | 0.05 | **0.02** | 0.4 (0.17-0.95) | **0.04** |
| **hsTnT admission:discharge** | >1.13408464 | 0.84 (0.36-1.95) | 0.69 | 0.89 | 0.84 (0.37-1.92) | 0.69 | 0.93 (0.39-2.19) | 0.86 | 0.63 | 0.92 (0.39-2.16) | 0.86 |
| **GDF-15 admission:discharge** | >1.8079528 | 0.26 (0.09-0.71) | **<0.01** | 0.18 | 0.26 (0.1-0.74) | **<0.01** | 0.25 (0.09-0.68) | **<0.01** | 0.15 | 0.25 (0.09-0.72) | **<0.01** |

**Supplementary Table S19** Survival analysis for 90-day all-cause mortality using continuous covariates

| **90-day all-cause mortality** | | | | | | |
| --- | --- | --- | --- | --- | --- | --- |
| **Factor** | **Univariate analysis** | | | **Multivariate analysis** | | |
|  | **Cox proportional hazards** | | **Schoenfeld residuals** | **Cox proportional hazards** | | **Schoenfeld residuals** |
|  | **HR (95%CI)** | **p-value** | **p-value** | **HR (95%CI)** | **p-value** | **p-value** |
| **Admission** | | | | | | |
| **Total orthoedema score** | 1.3 (0.62-2.75) | 0.48 | 0.17 |  |  |  |
| **Log NT-proBNP (pg/mL)** | 1.28 (0.83-1.98) | 0.25 | **0.048** | 0.88 (0.48-1.62) | 0.69 | **<0.01** |
| **Log hsTnT (pg/mL)** | 1.15 (0.84-1.59) | 0.41 | 0.28 | 1.13 (0.72-1.77) | 0.60 | 0.42 |
| **Log GDF-15 (pg/mL)** | 3.58 (1.24-10.29) | **<0.01** | 0.39 | 3.72 (1.19-11.7) | **<0.01** | 0.39 |
| **Discharge** | | | | | | |
| **Total orthoedema score** | 0 (0-Inf) | 0.26 | 1.00 |  |  |  |
| **Log NT-proBNP (pg/mL)** | 0.82 (0.5-1.34) | 0.42 | 0.31 | 0.69 (0.34-1.40) | 0.29 | 0.33 |
| **Log hsTnT (pg/mL)** | 0.89 (0.39-2.07) | 0.78 | 0.64 | 1.03 (0.32-3.28) | 0.96 | 0.66 |
| **Log GDF-15 (pg/mL)** | 1.41 (0.42-4.71) | 0.58 | 0.17 | 2.25 (0.46-11.0) | 0.30 | 0.15 |
| **Diff Log NT-proBNP (pg/mL)** | 1.08 (0.45-2.58) | 0.86 | 0.98 | 1.05 (0.35-3.15) | 0.93 | 0.70 |
| **Diff Log hsTnT (pg/mL)** | 0.71 (0.22-2.26) | 0.59 | 0.98 | 0.78 (0.15-4.04) | 0.77 | 0.66 |
| **Diff Log GDF-15 (pg/mL)** | 0.88 (0.11-6.81) | 0.90 | 0.17 | 0.94 (0.12-7.24) | 0.95 | 0.21 |

**Supplementary Table S20** Survival analysis for 90-day all-cause mortality using categorical variables

| **90-day all-cause mortality** | | | | | | | | |
| --- | --- | --- | --- | --- | --- | --- | --- | --- |
| **Factor** | **Threshold** | **Univariate analysis** | | | | **Multivariate analysis** | | |
|  |  | **Cox proportional hazards** | | **Log-Rank** | **Schoenfeld residuals** | **Cox proportional hazards** | | **Schoenfeld residuals** |
|  |  | **HR (95%CI)** | **p-value** | **p-value** | **p-value** | **HR (95%CI)** | **p-value** | **p-value** |
| **Admission** | | | | | | | | |
| **Grading orthoedema score** |  |  | 0.54 | 0.52 | 0.35 |  |  |  |
| No congestion | 0 | 1.00 (reference) |  |  |  |  |  |  |
| Low grade | 1-2 | 0.38 (0.04-3.7) | 0.41 | 1.00 |  |  |  |  |
| High grade | 3-4 | 0.81 (0.09-7.29) | 0.85 | 1.00 |  |  |  |  |
| **NT-proBNP** | >4577.00 pg/mL | 2.51 (0.51-12.42) | 0.23 | 0.25 | 0.08 | 1.63 (0.20-13.0) | 0.65 | **0.01** |
| **hsTnT** | >38.53 pg/mL | 0.92 (0.23-3.69) | 0.91 | 0.90 | 0.34 | 0.67 (0.10-4.43) | 0.68 | 0.69 |
| **GDF-15** | >6221.50 pg/mL | 541182300 (0-Inf) | **<0.01** | **0.01** | 1.00 | 505,667,633 (0.00-Inf) | 1.00 | 1.00 |
| **Discharge** | | | | | | | | |
| **Grading orthoedema score** | ≥1 (congestion present) | 0 (0-Inf) | 0.26 | 0.40 | 1.00 |  |  |  |
| **NT-proBNP** | >4577.00 pg/mL | 0.67 (0.06-7.35) | 0.74 | 0.74 | 0.14 | 0.97 (0.05-18.9) | 0.98 | 0.14 |
| **hsTnT** | >38.53 pg/mL | 0.58 (0.05-6.42) | 0.65 | 0.66 | 0.13 | 0.64 (0.04-11.7) | 0.77 | 0.13 |
| **GDF-15** | >6221.50 pg/mL | 0.62 (0.06-6.81) | 0.69 | 0.69 | 0.13 | 0.69 (0.06-8.46) | 0.77 | 0.13 |
| **NT-proBNP admission:discharge** | >1.0992464 | 2.08 (0.19-22.91) | 0.54 | 0.54 | 1.00 | 1.07 (0.07-17.2) | 0.96 | 0.16 |
| **hsTnT admission:discharge** | >1.13408464 | 0.51 (0.05-5.62) | 0.57 | 0.57 | 1.00 | 0.99 (0.06-16.0) | 0.99 | 0.16 |
| **GDF-15 admission:discharge** | >1.8079528 | 0.99 (0.06-15.76) | 0.99 | 0.99 | 0.16 | 0.99 (0.06-15.9) | 0.99 | 0.16 |

**Supplementary Table S21** Survival analysis for 180-day all-cause mortality using continuous covariates

| **180-day all-cause mortality** | | | | | | |
| --- | --- | --- | --- | --- | --- | --- |
| **Factor** | **Univariate analysis** | | | **Multivariate analysis** | | |
|  | **Cox proportional hazards** | | **Schoenfeld residuals** | **Cox proportional hazards** | | **Schoenfeld residuals** |
|  | **HR (95%CI)** | **p-value** | **p-value** | **HR (95%CI)** | **p-value** | **p-value** |
| **Admission** | | | | | | |
| **Total orthoedema score** | 1.04 (0.6-1.81) | 0.90 | 0.28 |  |  |  |
| **Log NT-proBNP (pg/mL)** | 1.22 (0.89-1.68) | 0.21 | 0.20 | 1.07 (0.70-1.63) | 0.75 | 0.17 |
| **Log hsTnT (pg/mL)** | 1.11 (0.86-1.44) | 0.44 | 0.43 | 1.05 (0.75-1.47) | 0.77 | 0.47 |
| **Log GDF-15 (pg/mL)** | 1.54 (0.87-2.73) | 0.13 | **0.02** | 1.44 (0.77-2.68) | 0.25 | **0.02** |
| **Discharge** | | | | | | |
| **Total orthoedema score** | 0.69 (0.24-1.95) | 0.44 | 0.48 |  |  |  |
| **Log NT-proBNP (pg/mL)** | 1.16 (0.85-1.57) | 0.34 | 0.13 | 1.2 (0.80-1.80) | 0.37 | 0.12 |
| **Log hsTnT (pg/mL)** | 1.08 (0.72-1.63) | 0.71 | 0.43 | 0.92 (0.52-1.62) | 0.77 | 0.40 |
| **Log GDF-15 (pg/mL)** | 1.16 (0.58-2.29) | 0.67 | 0.58 | 1.01 (0.45-2.26) | 0.98 | 0.58 |
| **Diff Log NT-proBNP (pg/mL)** | 0.65 (0.4-1.05) | 0.09 | 0.21 | 0.65 (0.38-1.10) | 0.12 | 0.33 |
| **Diff Log hsTnT (pg/mL)** | 0.7 (0.34-1.43) | 0.36 | 0.43 | 0.92 (0.41-2.06) | 0.84 | 0.36 |
| **Diff Log GDF-15 (pg/mL)** | 0.56 (0.2-1.56) | 0.27 | 0.69 | 0.78 (0.27-2.27) | 0.64 | 0.69 |

**Supplementary Table S22** Survival analysis for 180-day all-cause mortality using categorical variables

| **180-day all-cause mortality** | | | | | | | | |
| --- | --- | --- | --- | --- | --- | --- | --- | --- |
| **Factor** | **Threshold** | **Univariate analysis** | | | | **Multivariate analysis** | | |
|  |  | **Cox proportional hazards** | | **Log-Rank** | **Schoenfeld residuals** | **Cox proportional hazards** | | **Schoenfeld residuals** |
|  |  | **HR (95%CI)** | **p-value** | **p-value** | **p-value** | **HR (95%CI)** | **p-value** | **p-value** |
| **Admission** | | | | | | | | |
| **Grading orthoedema score** |  |  | 0.44 | 0.39 | 0.74 |  |  |  |
| No congestion | 0 | 1.00 (reference) |  |  |  |  |  |  |
| Low grade | 1-2 | 0.36 (0.07-1.8) | 0.21 | 1.00 |  |  |  |  |
| High grade | 3-4 | 0.59 (0.12-2.94) | 0.52 | 1.00 |  |  |  |  |
| **NT-proBNP** | >4577.00 pg/mL | 3.16 (0.88-11.33) | 0.05 | 0.06 | 0.48 | 4.13 (0.92-18.5) | 0.06 | 0.41 |
| **hsTnT** | >38.53 pg/mL | 0.91 (0.32-2.6) | 0.86 | 0.86 | 0.97 | 0.45 (0.13-1.57) | 0.21 | 0.98 |
| **GDF-15** | >6221.50 pg/mL | 2.15 (0.66-6.98) | 0.19 | 0.20 | **<0.01** | 1.77 (0.53-5.90) | 0.35 | **0.01** |
| **Discharge** | | | | | | | | |
| **Grading orthoedema score** | ≥1 (congestion present) | 0.48 (0.06-3.81) | 0.44 | 0.47 | 0.48 |  |  |  |
| **NT-proBNP** | >4577.00 pg/mL | 2.87 (0.72-11.47) | 0.12 | 0.12 | 0.24 | 4.27 (0.73-25.0) | 0.11 | 0.23 |
| **hsTnT** | >38.53 pg/mL | 1.45 (0.39-5.42) | 0.58 | 0.58 | 0.11 | 0.64 (0.12-3.29) | 0.59 | 0.13 |
| **GDF-15** | >6221.50 pg/mL | 0.99 (0.27-3.7) | 0.99 | 0.99 | 0.60 | 0.7 (0.18-2.76) | 0.61 | 0.59 |
| **NT-proBNP admission:discharge** | >1.0992464 | 0.53 (0.13-2.12) | 0.36 | 0.36 | 0.18 | 0.36 (0.07-1.77) | 0.21 | 0.28 |
| **hsTnT admission:discharge** | >1.13408464 | 1.26 (0.34-4.71) | 0.73 | 0.73 | 0.96 | 1.77 (0.42-7.46) | 0.44 | 0.62 |
| **GDF-15 admission:discharge** | >1.8079528 | 0.57 (0.14-2.37) | 0.43 | 0.43 | 0.81 | 0.54 (0.13-2.26) | 0.40 | 0.81 |

**Supplementary Table S23** Survival analysis for all-cause mortality using continuous covariates

| **All-cause mortality** | | | | | | |
| --- | --- | --- | --- | --- | --- | --- |
| **Factor** | **Univariate analysis** | | | **Multivariate analysis** | | |
|  | **Cox proportional hazards** | | **Schoenfeld residuals** | **Cox proportional hazards** | | **Schoenfeld residuals** |
|  | **HR (95%CI)** | **p-value** | **p-value** | **HR (95%CI)** | **p-value** | **p-value** |
| **Admission** | | | | | | |
| **Total orthoedema score** | 1.03 (0.64-1.64) | 0.92 | 0.46 |  |  |  |
| **Log NT-proBNP (pg/mL)** | 1.33 (1.01-1.76) | **0.03** | 0.85 | 1.31 (0.92-1.85) | 0.13 | 0.84 |
| **Log hsTnT (pg/mL)** | 1.08 (0.88-1.33) | 0.49 | 0.44 | 0.95 (0.72-1.25) | 0.69 | 0.47 |
| **Log GDF-15 (pg/mL)** | 1.44 (0.91-2.27) | 0.12 | 0.41 | 1.25 (0.76-2.05) | 0.38 | 0.42 |
| **Discharge** | | | | | | |
| **Total orthoedema score** | 0.92 (0.49-1.73) | 0.80 | 0.35 |  |  |  |
| **Log NT-proBNP (pg/mL)** | 1.18 (0.92-1.51) | 0.17 | 0.42 | 1.25 (0.91-1.71) | 0.17 | 0.43 |
| **Log hsTnT (pg/mL)** | 1.06 (0.77-1.47) | 0.72 | 0.89 | 0.89 (0.56-1.39) | 0.59 | 0.94 |
| **Log GDF-15 (pg/mL)** | 1.14 (0.68-1.93) | 0.62 | 0.99 | 1.0 (0.53-1.85) | 0.99 | 0.99 |
| **Diff Log NT-proBNP (pg/mL)** | 0.84 (0.57-1.25) | 0.40 | 0.31 | 0.87 (0.58-1.29) | 0.49 | 0.25 |
| **Diff Log hsTnT (pg/mL)** | 0.82 (0.41-1.65) | 0.58 | 0.42 | 0.95 (0.50-1.81) | 0.87 | 0.62 |
| **Diff Log GDF-15 (pg/mL)** | 0.64 (0.29-1.41) | 0.25 | 0.43 | 0.68 (0.31-1.52) | 0.33 | 0.49 |

**Supplementary Table S24** Survival analysis for all-cause mortality using categorical variables

| **All-cause mortality** | | | | | | | | |
| --- | --- | --- | --- | --- | --- | --- | --- | --- |
| **Factor** | **Threshold** | **Univariate analysis** | | | | **Multivariate analysis** | | |
|  |  | **Cox proportional hazards** | | **Log-Rank** | **Schoenfeld residuals** | **Cox proportional hazards** | | **Schoenfeld residuals** |
|  |  | **HR (95%CI)** | **p-value** | **p-value** | **p-value** | **HR (95%CI)** | **p-value** | **p-value** |
| **Admission** | | | | | | | | |
| **Grading orthoedema score** |  |  | 0.86 | 0.84 | 0.35 |  |  |  |
| No congestion | 0 | 1.00 (reference) |  |  |  |  |  |  |
| Low grade | 1-2 | 0.65 (0.14-2.9) | 0.57 | 1.00 |  |  |  |  |
| High grade | 3-4 | 0.72 (0.15-3.45) | 0.68 | 1.00 |  |  |  |  |
| **NT-proBNP** | >4577.00 pg/mL | 3.86 (1.30-11.49) | **<0.01** | **<0.01** | 0.91 | 6.15 (1.71-22.10) | **<0.01** | 0.82 |
| **hsTnT** | >38.53 pg/mL | 1.07 (0.45-2.53) | 0.87 | 0.88 | 0.59 | 0.41 (0.15-1.12) | 0.08 | 0.90 |
| **GDF-15** | >6221.50 pg/mL | 1.75 (0.70-4.4) | 0.22 | 0.23 | 0.30 | 1.30 (0.50-3.33) | 0.59 | 0.21 |
| **Discharge** | | | | | | | | |
| **Grading orthoedema score** | ≥1 (congestion present) | 0.85 (0.24-2.98) | 0.80 | 0.80 | 0.35 |  |  |  |
| **NT-proBNP** | >4577.00 pg/mL | 2.24 (0.80-6.32) | 0.12 | 0.12 | 0.75 | 3.17 (0.79-12.80) | 0.10 | 0.79 |
| **hsTnT** | >38.53 pg/mL | 1.43 (0.52-3.95) | 0.49 | 0.49 | 0.80 | 0.73 (0.19-2.83) | 0.65 | 0.73 |
| **GDF-15** | >6221.50 pg/mL | 0.89 (0.32-2.5) | 0.82 | 0.82 | 0.78 | 0.66 (0.22-1.96) | 0.45 | 0.77 |
| **NT-proBNP admission:discharge** | >1.0992464 | 0.74 (0.26-2.09) | 0.57 | 0.57 | 0.99 | 0.59 (0.20-1.75) | 0.34 | 0.82 |
| **hsTnT admission:discharge** | >1.13408464 | 1.24 (0.45-3.41) | 0.68 | 0.68 | 0.71 | 1.59 (0.55-4.63) | 0.39 | 0.92 |
| **GDF-15 admission:discharge** | >1.8079528 | 0.45 (0.15-1.36) | 0.15 | 0.15 | 0.64 | 0.42 (0.14-1.27) | 0.12 | 0.57 |
